# Thermoplasmonic Polymersome Membranes by *In Situ* Synthesis

**DOI:** 10.1021/acsnano.4c14093

**Published:** 2025-04-18

**Authors:** Valentino Barbieri, Javier González Colsa, Diana Matias, Aroa Duro Castano, Anshu Thapa, Lorena Ruiz-Pérez, Pablo Albella, Giorgio Volpe, Giuseppe Battaglia

**Affiliations:** 1 Department of Chemistry, University College London, 20 Gordon Street London WC1H 0AJ, United Kingdom; 2 Institute for Bioengineering of Catalunya (IBEC), The Barcelona Institute of Science and Technology (BIST), Barcelona 08028, Spain; 3 Group of Optics, Department of Applied Physics, University of Cantabria, Santander 39005, Spain; 4 Instituto de Medicina Molecular João Lobo Antunes (iMM), Lisbon 1649-028, Portugal; 5 Serra Húnter Fellow, Department of Applied Physics, University of Barcelona, Barcelona 08028, Spain; 6 Catalan Institution for Research and Advanced Studies (ICREA), Barcelona 08010, Spain

**Keywords:** hybrid polymersomes, thermoplasmonics, collective heating, cellular uptake, hyperthermia

## Abstract

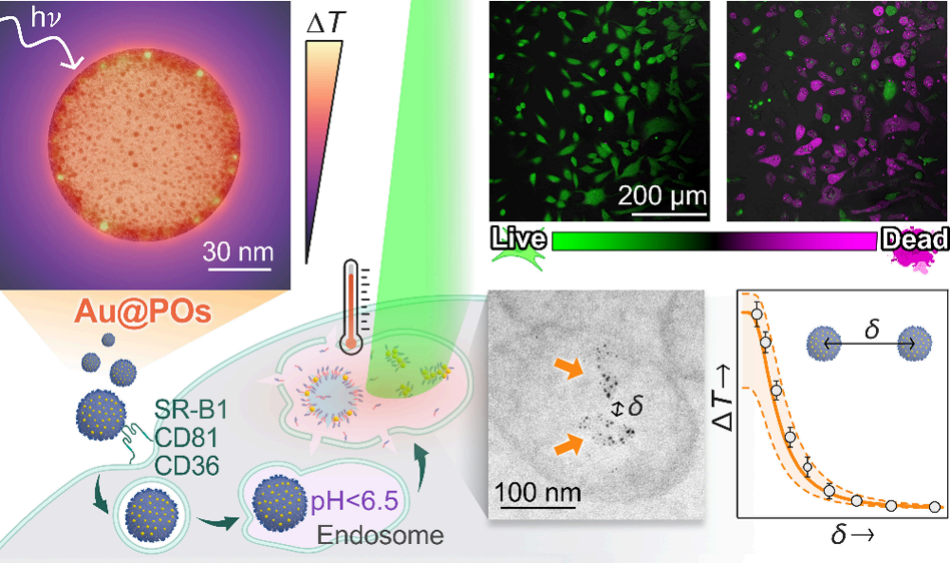

Thermoplasmonic nanoparticles, known for releasing heat upon illumination, find diverse applications in catalysis, optics, and biomedicine. Incorporating plasmonic metals within organic vesicle membranes can lead to the formation of nanoreactors capable of regulating temperature-sensitive microscopic processes. Yet, the controlled formation of stable hybrid vesicles displaying significant thermoplasmonic properties remains challenging. This work presents the *in situ* synthesis of highly efficient thermoplasmonic polymer vesicles, or hybrid polymersomes, by nucleating ∼2 nm gold nanoparticles within preformed polymersome membranes. This process preserves the vesicles’ morphology, stability, and overall functionality. Despite the small size of the embedded plasmonic nanoparticles, these hybrid polymersomes can efficiently convert laser light into a notable temperature increase on a larger scale through collective heating. We develop a theoretical framework that rationalizes the structure–property relations of hybrid polymersomes and accurately predicts their collective thermoplasmonic response. Finally, we demonstrate the biomedical potential of our polymersomes by employing their photothermal properties to induce the hyperthermal death of cancer cells *in vitro*, an effect amplified by their superior cellular uptake. We envision that these hybrid polymersomes will evolve into a versatile platform for precise control over nanoscale chemical and biological processes through plasmonic heating, unlocking numerous opportunities across various scientific and medical contexts.

## Introduction

Due to their ability to convert light into heat,^1^ plasmonic nanoparticles have attracted significant attention in physics and chemistry as a tool to enable remote control over diverse temperature-sensitive phenomena for applications in, e.g., sensing,^2^ imaging,^3,4^ optofluidics,^5^ and nanomedicine.^6^ The field of thermoplasmonics is particularly concerned with the development, characterization, and optical modeling of plasmonic nanoparticles for their use as heat sources remotely controlled by light.^7^ Hybrid structures that combine plasmonic metals, e.g., gold nanoparticles, with organic materials represent a promising avenue to synthesize nanoreactors with temperature control over chemical and biological processes at the micro- and nanoscales. For example, the surface of gold nanoparticles can be functionalized with amphiphilic polymers to induce their self-assembly into vesicular structures and tailor their plasmonic response to various theranostic applications.^8^

Incorporating small plasmonic nanoparticles directly within the membranes of soft organic vesicles is key to synthesizing such hybrid systems without compromising key vesicle functionalities, such as their colloidal stability, external surface chemistry, and capability to carry additional payloads in their lumen.^9^ Polymersomes synthetic vesicles made of amphiphilic block copolymers—are established nanocarriers that can be designed to boast tailored properties, including enhanced stability, bioavailability, and high chemical versatility.^10−13^ These features make them highly suitable for applications in nanomedicine, such as targeted delivery.^14−18^

To date, hybrid polymersome membranes have mainly been created by enclosing preformed inorganic nanoparticles during the polymersome self-assembly process.^9,19−25^ However, this process requires tight control of conditions and imposes modest limits on nanoparticle loading to prevent alterations in the final structure, stability, or nanocarrier functionality compared to pristine vesicles.^26^ A few studies have reported the *in situ* synthesis of gold nanoparticles within polymersome membranes for nonplasmonic purposes, such as imaging contrast agents,^27^ catalysis,^28^ or tuning drug release profiles.^29^ Yet, due to the scarce investigation of the plasmon resonance in these systems, it remains unclear whether embedding small nanoparticles in the membrane by *in situ* synthesis can yield tangible thermoplasmonic properties.^28,30^

In this work, we present functional thermoplasmonic polymersomes produced via the *in situ* synthesis of 2 nm gold nanoparticles within the membranes of pH-sensitive poly[(2-methacryloyl)ethyl phosphorylcholine]_25_-*block*-poly[2-(diisopropylamino)ethyl methacrylate]_80_ (PMPC–PDPA) polymersomes. This synthesis does not affect the morphology or colloidal stability of polymersomes compared to their pristine counterparts. After characterizing their morphology and gold loading capability, we demonstrate the efficient thermoplasmonic properties of the newly synthesized hybrid polymersomes. In addition, we develop a theoretical model to explain the structure–function relationship in the photothermal response of our system. Finally, we demonstrate that the hybrid polymersomes’ thermoplasmonic response can be maximized via collective effects upon accumulation within cancer cells via receptor-mediated endocytosis, resulting in hyperthermal cell killing.

## Results and Discussion

### Synthesis of Thermoplasmonic Polymersomes

**Figure 1:**
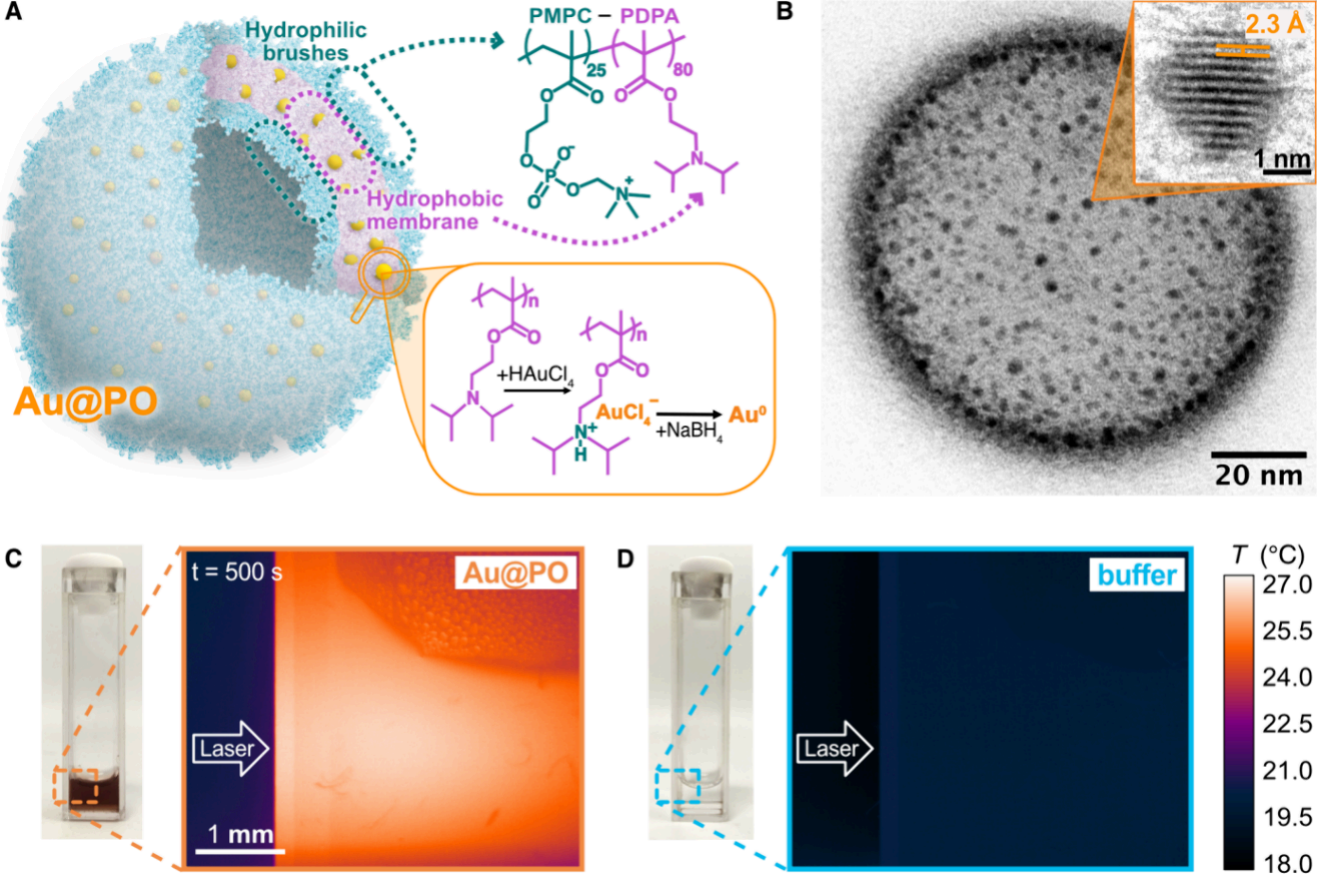
The design concept of thermoplasmonic hybrid polymersomes. (A) Schematic of a hybrid polymersome (Au@PO). Amphiphilic PMPC–PDPA block copolymer molecules self-assemble into polymersomes in aqueous environments. Gold nanoparticles (AuNPs) are nucleated within the PDPA membrane via the *in situ* reduction of chloroauric acid (HAuCl_4_) to metallic Au^0^. (B) Representative transmission electron microscopy (TEM) image of a hybrid polymersome. AuNPs are detectable as dark dots due to their high electron density. The TEM specimen was positively stained with phosphotungstic acid to visualize the polymer matrix. Inset: high-resolution TEM image of a single gold nanoparticle showing the spacing between the Au(111) lattice planes. (C, D) Infrared images showing the temperature increase from (C) an Au@PO dispersion (∼6 Au@POs μm^–3^) in 0.1 M phosphate buffer and (D) the buffer alone upon exposure to a 532 nm laser with a power density of 0.128 mW μm^–2^. The arrows indicate the point of incidence of the laser.

We fabricated biocompatible hybrid polymersomes consisting of PMPC–PDPA vesicles embedding randomly distributed gold nanoparticles within their membrane (Figure 1A). The membrane-forming PDPA block contains pH-sensitive tertiary amine groups capable of coordinating gold ions and templating their crystallization *in situ*, thus spatially confining the nucleation of gold nanoparticles inside the membrane.^29^ Concurrently, the hydrophilic brushes impart bioavailability to the particles, enabling us to showcase their activity in biological environments. The zwitterionic polymer PMPC is well known for its antifouling properties.^31−33^ Hence, it is expected to minimize undesired nonspecific interactions when polymersomes are immersed in crowded biological environments. In previous studies, we showed that PMPC–PDPA polymersomes can transport various types of cargo into cells through receptor-specific targeting. These include nucleic acids, small molecules, proteins, and even small nanoparticles. In particular, we demonstrated that the phosphorylcholine motif contained in PMPC targets three cell receptors: SR-B1, CD36, and CD81.^34−36^ Exploiting this interaction, PMPC enables the targeted delivery to either cancer cells or myeloid cells.^16,37,38^ Our extensive body of prior research demonstrates that these polymersomes can navigate biological fluids rich in serum proteins while maintaining the stability and surface functionalities necessary for efficient uptake in target cells. In particular, our previous investigations of protein corona formation on the surface of PMPC–PDPA polymersomes indicate no detectable interactions between common plasma proteins and polymersomes.^39^ Our current approach aims to prevent protein corona-induced aggregation by nucleating gold nanoparticles within the polymersome membrane, where they are shielded from direct interactions with the external environment by the protein-repelling PMPC brush.

In this work, we produced PMPC–PDPA polymersomes by the “solvent switch” method. This process triggers the bottom-up assembly of amphiphilic block copolymers into supramolecular structures by gradually exchanging an organic solvent that dissolves both blocks for a buffered aqueous solution (selective for the PMPC block only). Polymersome dispersions with average diameters between 45 and 100 nm were obtained depending on the process conditions. We then proceeded to the *in situ* synthesis of the gold nanoparticles within the polymersome membranes. The initial step of this reaction involves the partial protonation of PDPA membranes by the acidic gold precursor HAuCl_4_. In this step, polymersomes were dispersed in phosphate saline buffer at pH 7.4 and continuously stirred in an ice bath. The buffer’s pH is slightly lower than the polymer’s p*K*_a_ ≈ 7.6 at ice bath temperatures. As a result, extensive protonation (over 60%) is attained, while the reduced mobility of entangled high-molecular-weight polymers at low temperatures preserves the polymersome integrity.^40^ Upon protonation, the positively charged polymersome membrane attracts the AuCl_4_^–^ anions, which diffuse to the membrane and interact with the amines in the PDPA block. After adding the NaBH_4_ reducing agent, the nucleation of metallic Au seeds occurs, and their growth remains templated within the polymer membranes.^27^

Transmission electron microscopy (TEM) imaging confirmed the successful outcome of the reaction, as exemplified in Figure 1B. To visualize the polymer assembly, we stained the specimen with phosphotungstic acid (PTA), which acts as positive staining on PMPC–PDPA.^41^ The resulting image presents a “chocolate-chip cookie” appearance, in which gold nanoparticles appear as dark dots included within the polymersome membrane organic matrix. High-resolution TEM imaging (Figure 1B, inset) of the embedded gold nanoparticles reveals the crystalline lattice planes from which the crystallographic structure could be assigned. The measured interplanar spacing of (0.23 ± 0.01) nm is in good agreement with the reported value for Au(111) planes in the face-centered cubic unit cell commonly adopted by plasmonic sub-5 nm gold nanoparticles.^42,43^ TEM analysis also shows that the growth of gold nanoparticles does not create gaps or voids in the PDPA membrane compared to pristine polymersomes (Figure S1). Such a continuous embedment can be ascribed to the active role of the PDPA block in templating the gold nanoparticles’ nucleation. Additionally, ζ-potential measurements show no variation in surface charge upon the growth of gold nanoparticles. The surface potential distributions reported in Figure S2 remained centered at neutral values, thus confirming that the surface interactions are dominated by the intrinsically neutral zwitterionic PMPC polymer brush.

Infrared imaging confirms that the hybrid polymersome dispersion can dissipate the radiation absorbed when illuminated with a 532 nm laser in the form of heat (Movie S1). Figure 1C and Figure 1D show the spatial temperature distributions reached in the dispersion and the control, respectively, after 500 s of illumination. While the hybrid polymersome dispersion (Figure 1C) generated a net increase in temperature at the laser incidence spot, the buffer alone (Figure 1D) did not present any detectable deviation from room temperature.

### Morphological Characterization

In order to explore the relationship between the amount of gold loaded in our system and its impact on the thermoplasmonic response, we produced hybrid polymersomes at varying molar Au/DPA ratios (measured in mol %). The term “DPA” refers to the 2-(diisopropylamino)ethyl methacrylate repeating unit of the PDPA block. Within the concentration range of 3–25 mol %, the reaction produced hybrid polymersomes with substantially unchanged size distributions and colloidal stabilities compared to the pristine (i.e., nondecorated) polymersome dispersions, as observed by dynamic light scattering (DLS) measurements (Figure S3). Further addition of HAuCl_4_ to achieve Au/DPA ratios above 25 mol % decreased the pH below 7, severely affecting the stability of hybrid polymersomes. While structural alterations are kinetically hindered at low temperatures, significant negative deviations from the p*K*_a_ are known to trigger polymersome disassembly.^40^

**Figure 2:**
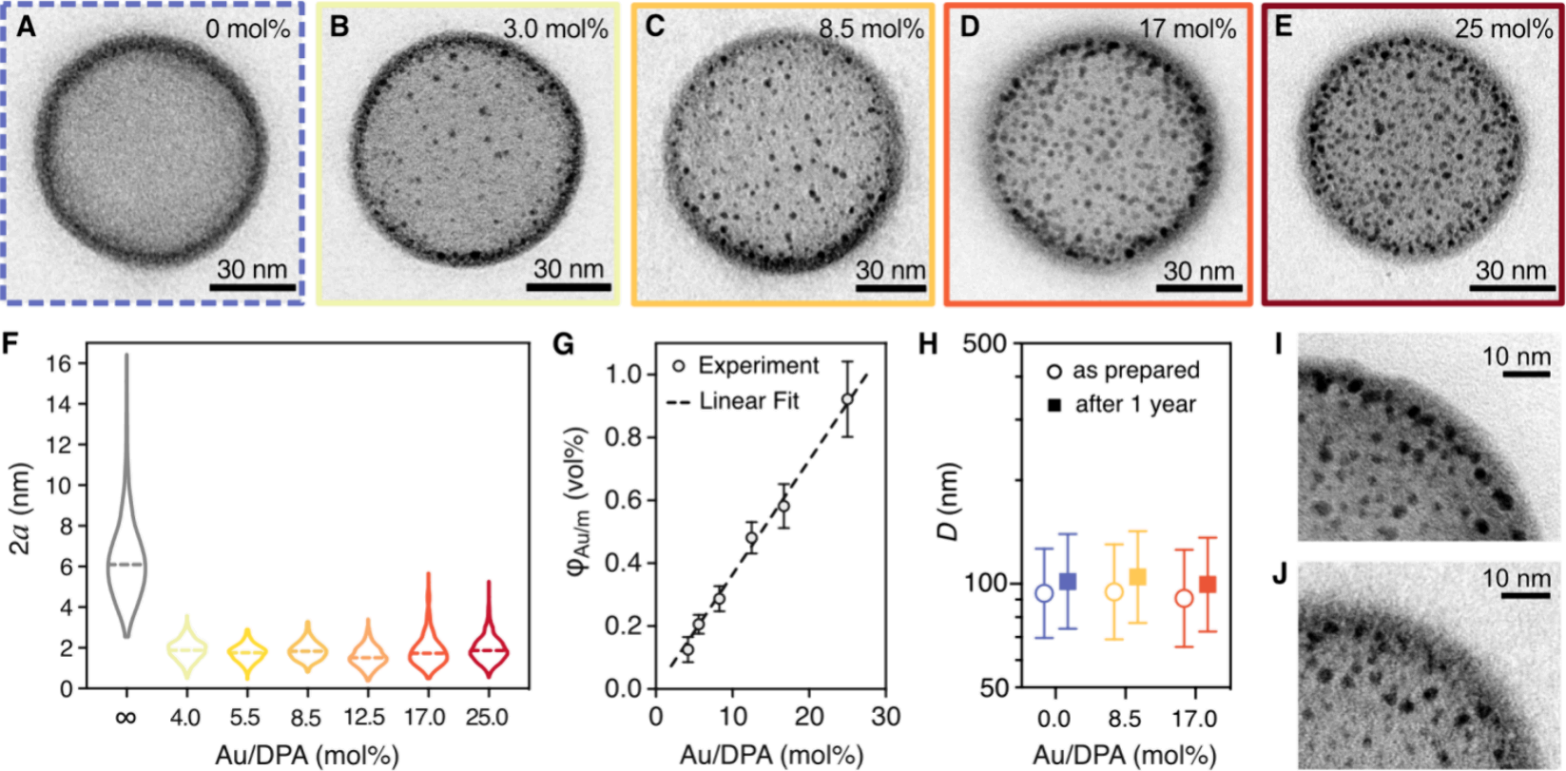
Morphological characterization of hybrid polymersomes. (A–E) High-magnification TEM images of (A) pristine polymersomes and (B–E) hybrid polymersomes synthesized at increasing gold-to-monomer molar ratios (Au/DPA): (B) 3 mol %, (C) 8.5 mol %, (D) 17 mol %, and (E) 25 mol %. The images illustrate the average Au loading at varied Au/DPA ratios. Representative images showing the variability in loading capability are shown in Figures S6 and S7. (F) Violin plots representing the diameter (2*a*) distributions of the gold nanoparticles embedded in the polymersome membranes as a function of the Au/DPA mol %. The ∞ mol % corresponds to free gold nanoparticles synthesized without polymersomes. Each violin plot represents the distributions of >450 gold nanoparticles as measured from TEM images; dashed lines mark the medians. (G) Linear relationship between the Au loading ratio φ_Au/m_ and the initial Au/DPA molar ratio fed into the reaction. The error bars represent the error calculated from three repeated concentration measures per sample. (H) Stability of the hybrid polymersome diameters *D* over a year of storage at 4 °C. The means (symbols) and standard deviations (error bars) of the diameter distributions measured by DLS display negligible changes for both pristine and hybrid polymersomes. (I, J) Details of TEM images providing a magnified view of the gold nanoparticles embedded within hybrid polymersomes (I) immediately after synthesis and (J) after a year stored at 4 °C.

TEM analysis of hybrid polymersomes, exemplified by the images in Figure 2A–E, confirms that polymersomes retained the spherical morphology upon increased Au concentration and validates the accuracy of DLS sizing. Additional examination of the TEM images suggests that the final gold nanoparticle size remains constant while the average loading density rises with the Au/DPA ratio. The uniformity in size and shape of embedded gold nanoparticles can be better appreciated, alongside their spatial arrangement and crystalline structure, in the unstained TEM images of hybrid polymersomes shown in Figure S4.

We further confirmed their intersample uniformity by sizing over 450 particles from images of various hybrid polymersomes in each formulation (Figure 2F). The diameters of the embedded gold nanoparticles remain narrowly distributed around the mean value of 1.9 ± 0.5 nm as the Au/DPA ratio increases, consistent with their templated growth within the polymersome membrane. Conversely, when gold nanoparticles are synthesized under the same conditions but without polymersomes (Au/DPA → ∞ in Figure 2F), significantly larger and more broadly distributed diameters were measured immediately after the reduction. Such bare nanoparticles showed colloidal instability and eventually precipitated.

To characterize the Au loading quantitatively, we measured the Au and polymer concentrations in our formulations by microwave-assisted plasma atomic emission spectroscopy (MP-AES) and high-performance liquid chromatography (HPLC), respectively. Combining the concentrations with morphological information (“Au Loading Estimation” Section in the Supporting Information), we defined the membrane loading volume ratio, φ_Au/m_, as the ratio between the volume fractions of Au and the PDPA membranes in the samples. Figure 2G shows a linear relationship between φ_Au/m_ and the Au/DPA molar ratio fed into the reaction. An analogous trend is observed when the loading ratio is expressed as output Au/DPA mol % in Figure S5. The slope of this second fit indicates an 87% yield of Au loading using our protocol. The plots include data from 24 distinct formulations synthesized from five different pristine polymersome batches. The strong linearity across such a diverse set of formulations highlights the precise control over the final hybrid polymersome morphology achieved through the *in situ* reaction. The final volume of gold embedded in our hybrid polymersomes’ membranes can be determined just by acting on a single variable, i.e., the HAuCl_4_ precursor concentration. Note that the loading ratio should be intended as an average across the whole polymersome population. Low-magnification TEM observations revealed that gold nanoparticles are unevenly distributed among polymersomes at low Au concentrations, with only some of the imaged polymersomes being decorated and others remaining empty (Figure S6). Only above 12.5 mol % of Au/DPA were all the polymersomes in the sampled population found to display hybrid membranes (Figure S7).

We finally assessed the long-term stability of our hybrid polymersomes by repeating the morphological characterization of three formulations after 1 year of storage at 4 °C. Both the pristine and hybrid polymersomes exhibited outstanding stability over time, as confirmed by DLS analysis in Figure S8A,B. No signs of degradation or instability, such as oscillations or peaks at long lag times, were observed in the autocorrelation functions presented in Figure S8A. The size distributions displayed in Figure S8B remained unchanged over 1 year for all three tested formulations, with the curves shifting by less than the overall uncertainty of the measure (95% confidence interval of the mean). The variation in the mean polymersome diameters (symbols in Figure 2H) was minimal compared to the width of the distributions (error bars) and unaffected by the loading ratio of gold nanoparticles. Concurrently, the polymersome populations shown in the low-magnification TEM images of Figure S8C–E align nicely with our DLS analysis. TEM imaging conducted at higher magnification before (Figure S8F) and after (Figure S8G) long-term storage confirms the preservation of the hybrid polymersome morphology over time. Finally, the close-up TEM images in Figure 2I,J indicate that long-term storage did not affect the size or integration of gold nanoparticles within the membranes of hybrid polymersomes.

### Thermoplasmonic Characterization and Modeling

**Figure 3:**
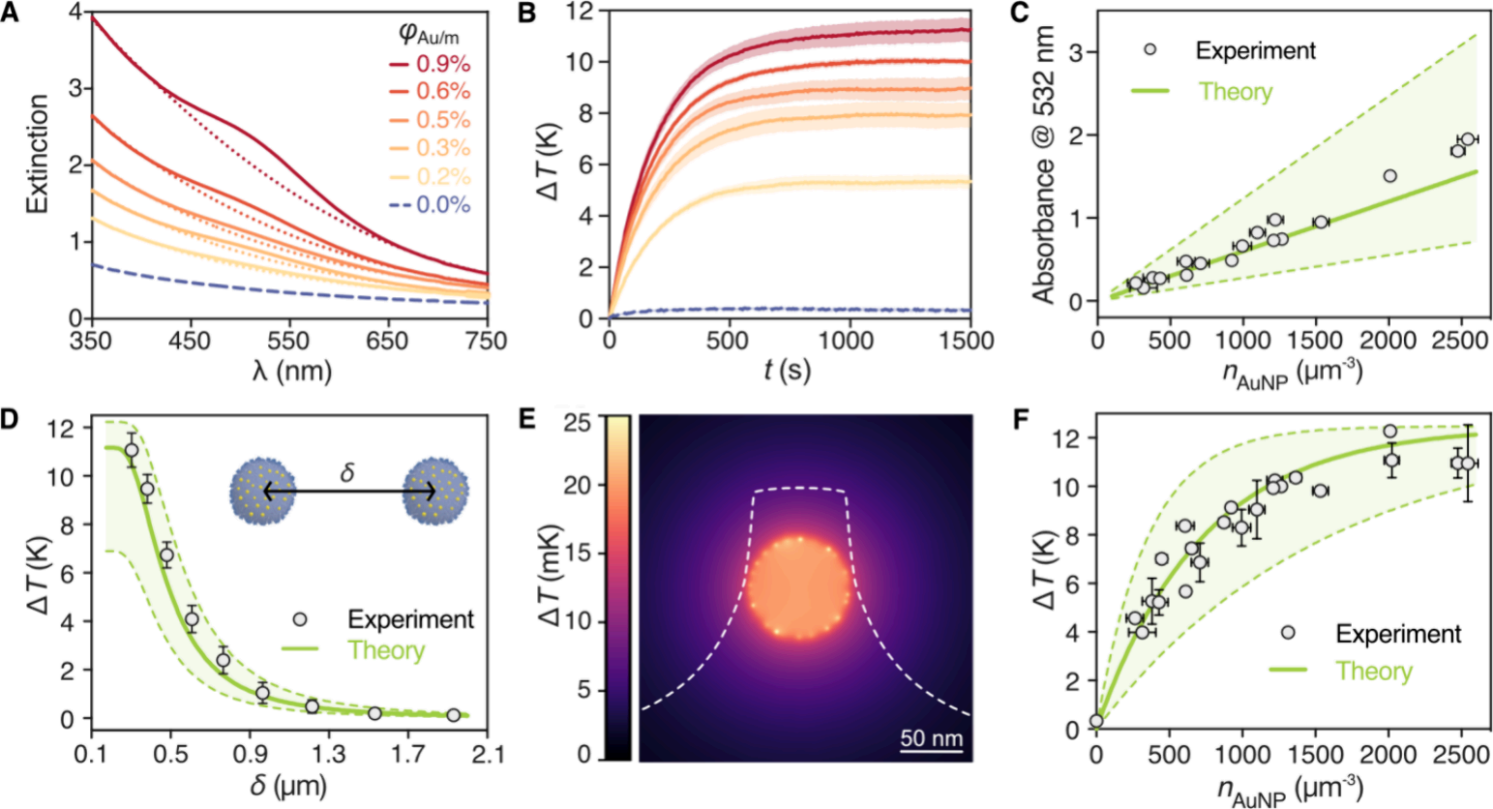
Thermoplasmonic properties of hybrid polymersomes. (A) Extinction spectra (solid lines) of hybrid polymersome dispersions of increasing loading ratio φ_Au/m_. The spectrum of pristine polymersomes (dashed line) is shown for reference. Dotted lines represent polynomial fits of the UV and NIR tails. (B) Temperature evolution (solid lines) of hybrid polymersome dispersions with increasing φ_Au/m_ under 532 nm laser exposure (*I* = 0.128 mW μm^–2^). The profile of pristine polymersomes (dashed line) is shown for reference. Shaded areas indicate the standard error from five measurements across three formulations. (C) Absorbance at 532 nm varies linearly with the number density of gold nanoparticles, *n*_AuNP_. Each data point univocally represents the absorbance of an individual sample. Horizontal error bars reflect uncertainties in *n*_AuNP_ due to propagated experimental errors. (D) Experimental steady-state temperature increments Δ*T* (circles) under the same laser exposure as in (B) for a progressively diluted dispersion of 100 nm hybrid polymersomes (φ_Au/m_ = 0.9 vol %, χ_AuNP_ = 262) plotted against mean interparticle spacing, δ (illustrated in the inset). Data points are means of three measurements, with error bars showing standard deviation. (E) Simulated temperature map of a single 100 nm hybrid polymersome under the same conditions as (D). The overlaid dashed white line represents the angle-averaged radial temperature profile. (F) Experimental Δ*T* (circles) from all tested dispersions under laser exposure as a function of *n*_AuNP_. Each data point represents an individual sample. The corresponding temperature traces are shown in Figure S10. In (C, D, F), theoretical predictions are shown as solid green lines. Confidence interval boundaries (dashed lines) are estimated using cross sections calculated at the limits of gold nanoparticle and polymersome size distributions.

As demonstrated in the preliminary experiment of Figure 1C,D, our hybrid polymersomes exhibit a macroscopically measurable thermoplasmonic response. This phenomenon originates from the resonant absorption of incident photons by the gold nanoparticles embedded in the polymersome membrane. Figure 3A shows the extinction spectra of hybrid polymersome dispersions as a function of their Au loading ratio φ_Au/m_. While the extinction of pristine polymersomes (dashed line) monotonically decays as the incident wavelength increases across the visible spectral region, the localized surface plasmon resonance (LSPR) appears as a broad feature centered around 520 nm in the spectra of hybrid polymersomes. Our observation of an LSPR feature in hybrid polymersomes produced by the *in situ* synthesis differs from the findings of Zhu et al.,^28^ who suggested that the absence of LSPR could result from the successful immobilization of the gold nanoparticles in the polymer matrix. Our results demonstrate, instead, that plasmonic properties are compatible with the *in situ* nucleation of small nanoparticles embedded in the membranes. To better isolate and analyze the absorption features, polynomial decay fits joining the UV and NIR tails of the spectra are plotted as dotted lines. The flattened and broad shape of the LSPR is typical of ultrasmall (<5 nm) nanoparticles, owing to the damped electron vibrations at such high levels of confinement.^44,45^ In addition, Figure 3A shows that the LSPR prominence increases with the loading ratio of Au in the membrane.

We then assessed the thermoplasmonic heat generation in hybrid polymersomes dispersions. We utilized an internally developed and calibrated resistance thermal probe to precisely collect the temperature evolution at the center of a quartz cuvette containing the samples under exposure to a 532 nm laser (Figure S9A). To evaluate the contribution of the experimental setup to the temperature increase, we applied an equivalent stimulus to a cuvette containing only the dispersion buffer (Figure S9B). The resulting equilibrium temperature increase of 0.14 K was comparable to room temperature fluctuations. This value was subtracted from the reported temperatures of pristine and hybrid polymersome suspensions.

Next, we applied an illumination history consisting of sequentially increasing laser power steps to a hybrid polymersome dispersion of fixed φ_Au/m_ (Figure S9C). Within each step, we observed that the temperature near the focal spot grew sharply in the first transient, leveling off as the heat was transferred to the surroundings through convection and conduction. After ≈600 s of exposure, the whole sample approached a thermal equilibrium plateau that was maintained until we increased the power density again. When, at last, we removed the stimulus, the temperature dropped to room temperature. Notably, the equilibrium temperature grew proportionally with the incident laser power density (Figure S9D).

In Figure 3B, we report the temperature increment profiles recorded for dispersions of hybrid polymersomes with increasing gold loading at a fixed power density of 0.128 mW μm^–2^. The traces, averaged over φ_Au/m_ from those presented in Figure S10A, follow the previously described saturation trend and reach higher steady-state temperature increments as gold loading increases. This result further supports the thermoplasmonic nature of the phenomenon, as heat emission follows from resonant light absorption, which we also showed to increase with gold loading.

In addition, we confirmed the remarkable structural stability of our hybrid polymersomes under repeated laser stimulation. TEM inspection of a hybrid polymer dispersion after three consecutive thermoplasmonic heating–cooling cycles revealed no noticeable morphological changes compared to the untreated sample, as shown in Figure S11.

To rationalize the concentration dependence arising from the collective thermoplasmonic excitation of our hybrid polymersomes, we developed an ad hoc theoretical model (refer to the SI for complete development). We started by obtaining the optical cross sections of the two components of our hybrid system independently. The theory suggests that scattering is negligible for gold nanoparticles, and absorption is negligible in polymersomes at all sizes in our distributions. Therefore, the extinction cross sections of each hybrid polymersome can be expressed as the linear superposition of the scattering cross section of the polymer shell, σ_sca_, and the absorption cross sections σ_abs_ of all the gold nanoparticles embedded in its membrane:
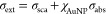
1

Here, χ_AuNP_ represents the loading capacity of hybrid polymersomes, defined as the average number of gold nanoparticles per polymersome. To compute these cross sections, we modeled the hybrid vesicle as a four-layer stratified sphere with a water core surrounded by three concentric polymer layers: a PDPA layer containing the gold nanoparticles sandwiched between two hydrated PMPC layers (Figure S12). We computed the extinction cross sections of polymersomes with different diameters by applying Mie theory to this geometry. For the optical properties of our ultrasmall (∼2 nm) gold nanoparticles, we calculated the Mie solutions in the quasi-static approximation; to account for additional plasmon confinement effects, we integrated a semiempirical extension of the Drude model proposed by Karimi et al.^45^ Since polymersomes account entirely and exclusively for scattering, the experimental absorption of each hybrid polymersome dispersion was obtained by subtracting the extinction of the parent pristine polymersome dispersion at equivalent number density *n*. In Figure 3C, we show that the absorbance in the LSPR region—here at 532 nm—grows linearly with the number density of gold nanoparticles.

We plotted theoretical prediction lines calculated through Karimi’s model^44^ for the average (solid lines) and boundaries (dashed lines) of our experimental size distribution of gold nanoparticles. Given the excellent match, we conclude that the theoretical model accurately describes the optical behavior of our *in situ*-grown gold nanoparticles. Hence, eq 1 was used to model the optical response of hybrid polymersomes in our thermoplasmonic model.

Upon illumination with a laser of intensity *I*, absorption events in a homogeneous dispersion of hybrid polymersomes generate a local heat flow rate in the infinitesimal volume d*V*:
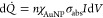
2where *n* is the number density of the hybrid polymersomes and χ_AuNP_ their average loading capacity. The laser intensity is attenuated along the propagation axis *z* by absorption and scattering phenomena, which depend on concentration according to the Lambert–Beer law:
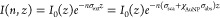
3

For a Gaussian beam of incident power *P* and cross-sectional area *A*(*z*), the intensity can be expressed as , so that the integration of eq 2 over the illuminated volume gives
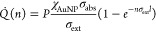
4

where *l* is the path length. After heat generation, thermal energy is exchanged with the external environment through convective flows along the boundaries and conduction through the cuvette walls. An overall heat transfer coefficient β = *Q̇*(*n*)/Δ*T* can account for both phenomena (SI). The resulting theoretical steady-state temperature increment Δ*T* can be calculated as
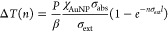
5

To validate our model, we selected a hybrid polymersome dispersion with φ_Au/m_ = 0.9 vol % and an average diameter of 100 nm, yielding χ_AuNP_ = 260. This morphology ensures significant contributions from both scattering and absorption. We measured Δ*T* in the dispersion under illumination at a constant incident power density (0.128 mW μm^–2^), gradually decreasing the number density *n* through consecutive dilutions (Figure S13). The curves obtained by evaluating eq 5 at the center (solid line) and boundaries (dashed lines) of the sample size distributions are plotted for comparison. Our theoretical predictions accurately describe the data (*R*^2^ = 0.97), with all experimental points falling within the calculated confidence interval. By applying a change of variable (see eq S22), we replotted the same data as a function of the mean interparticle distance δ among neighboring hybrid polymersomes (Figure 3D). This representation enables us to examine the collective nature of the phenomenon by directly comparing the δ-dependence of the dispersion’s global temperature with the thermal field around individual polymersomes, as simulated by finite element modeling. Figure 3E illustrates the spatial temperature distribution generated by an isolated hybrid polymersome in its surroundings under analogous conditions to the experiment of Figure 3D. The temperature reaches a maximum of 25 mK on the surface of the embedded gold nanoparticles, from where heat diffuses into the polymer layers and the aqueous medium inside and outside the polymersomes. A uniform temperature of 20 mK is maintained in the polymersome core due to the spatial confinement of the warm liquid. Externally to the polymersome, the temperature decreases inversely with the radial distance, reaching only 1.7 mK at 200 nm from the center. Hence, as exemplified in Figure 3D, the spatial proximity between polymersomes is paramount for their individual temperature fields to overlap and build up to macroscopic global temperatures able to affect chemical and biological processes. Indeed, our theory predicts that proximity-driven collective effects can have a stronger influence on the thermoplasmonic temperature than gold loading. The calculated Δ*T* for different volume fractions φ_Au/m_ (Figure S14) drops sharply below 1 K as interpolymersome distances increase within a range of less than 10 diameters. In contrast, an almost 80% reduction in gold loading results in a temperature drop of less than 3 K in highly concentrated dispersions.

Our model can be further extended to analyze the laser-induced temperature evolution in all 22 hybrid polymersome formulations tested as a function of the gold nanoparticle number density alone (see Figure S10 for individual time–temperature traces). This simplification can be introduced by noting that, for hybrid polymersomes with a diameter of less than 100 nm, the scattering cross section is at least one order of magnitude lower than the collective absorption of the embedded gold nanoparticles at any tested loading capacities. Therefore, the dissipation introduced by scattering can be neglected without losing accuracy. This leads to an alternative expression for eq 5 (from eq S23):
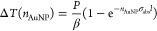
6

This generalization eliminates the dependence of the temperature on the polymersome morphology and allows us to predict the experimental trend emerging from all our data points analytically (Figure 3F). Once again, the experimental Δ*T* values are all contained within the theoretical confidence interval defined over the gold nanoparticle size distribution, confirming the robustness (*R*^2^ = 0.92) of our theoretical model.

### Hybrid Polymersomes as Intracellular Heaters: Application in Hyperthermia

**Figure 4:**
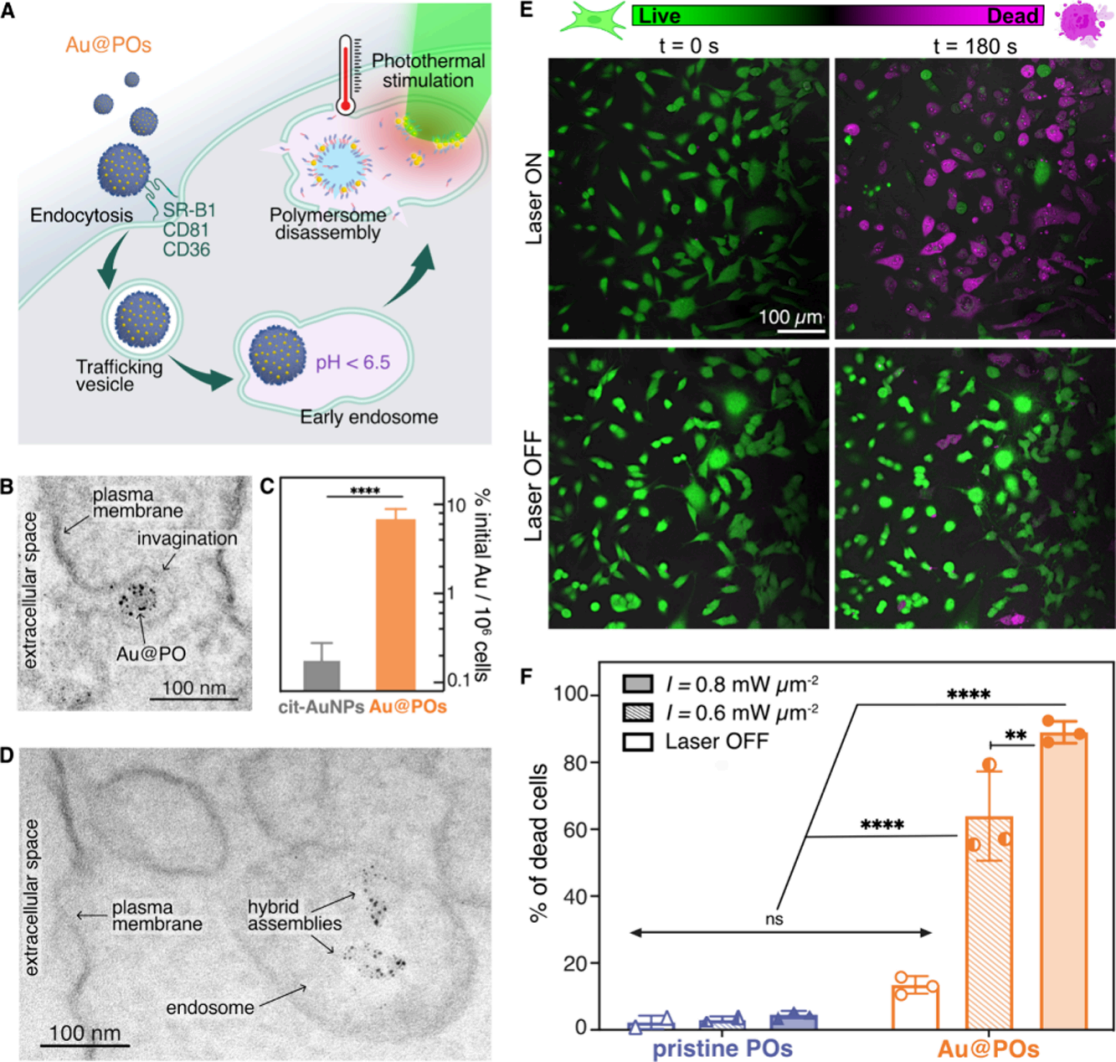
Plasmonic hyperthermia with hybrid polymersomes. (A) Schematic of the proposed intracellular activation mechanism of laser-induced hyperthermia with hybrid polymersomes (Au@POs). Left to right: Au@POs interact with the SR-B1, CD36, and CD81 receptors expressed on the surface of cells, promoting endocytosis. Au@PO is transported via a trafficking vesicle to the endosome, where protonation of the pH-sensitive PDPA membrane drives the partial degradation of Au@POs and hybrid assemblies accumulate in the endosome. Laser illumination stimulates a localized temperature increase that induces cell death. (B) TEM image of Au@POs being endocytosed by a T98G human glioblastoma cell after 1 h of incubation. A representative event is marked in the image. (C) The Au mass uptake by T98G cells after 1 h of incubation with Au@POs shows an almost 40-fold higher uptake than with citrate-stabilized gold nanoparticles (cit-AuNPs). Error bars are the standard deviations of four (cit-AuNPs) and eight (Au@POs) independent experiments. (D) TEM image of hybrid Au-polymer assemblies within an endosome of a T98G cell. (E) Live/dead analysis of T98G cells treated with Au@POs and exposed to a 514 nm confocal microscope laser. Fluorescence microscopy composite images of cells labeled with a live/dead fluorescent staining, with emissions represented in green (live) and magenta (dead). The images show the death of cells treated with Au@PO after 3 min at 0.8 mW μm^–2^ (top row). Cells treated with Au@POs without laser exposure (bottom row) show a significantly reduced death rate. (F) Mortality of T98G cells treated with Au@PO or pristine polymersomes (POs) and exposed for 3 min to a 514 nm scanning laser at *I* = 0.8 mW μm^–2^ (solid), *I* = 0.6 mW μm^–2^ (striped), and without laser exposure (clear) as determined by live/dead assays. Data points represent independent experiments; columns represent means, and error bars are standard deviations. Statistical significance was evaluated by Student’s *t* test in (C) and two-way ANOVA in (F), with ns *p* > 0.05, **p ≤ 0.01, and ****p ≤ 0.0001.

We have so far demonstrated that hybrid polymersomes produced by the *in situ* Au-nucleation reaction possess excellent morphological uniformity and stability. Furthermore, the proposed system can produce plasmonic heat that can be used to influence biological processes. Harnessing plasmonic resonances has already enabled applications in various areas of nanomedicine, including biosensing, imaging, and photothermal therapy.^46−48^ The latter is a promising noninvasive approach for treating solid tumors relying on laser-induced hyperthermia to destroy cancer cells selectively, thus minimizing the adverse effects commonly associated with traditional cancer treatments.^49^ Based on our previous work,^37,38^ we expect that PMPC–PDPA will facilitate the cell internalization of the hybrid assemblies, following the pathway exemplified in Figure 4A. The phosphorylcholine moiety of the PMPC block in the brush is known to selectively bind to a subset of receptors overexpressed on many different cell lines: SR-B1, CD36, and CD81.^16,50−53^ Such affinity promotes the endocytosis of hybrid polymersomes.

Direct evidence of the endocytic event was obtained through TEM observation of human glioblastoma cells of the T98G line, treated *in vitro* with 100 nm hybrid polymersomes (Figure 4B). These cells express the two main PMPC target receptors, i.e., SR-B1 and CD36, as demonstrated by Western blot analysis on T98G cell lysates (Figure S15). We quantified the cellular uptake of gold in T98G cells by MP-AES after 60 min of incubation with our formulations and thorough washing of the unbound particles. The cellular uptake of Au from receptor-binding hybrid polymersomes was nearly 40 times more efficient than from nontargeting gold nanoparticles (cit-AuNPs) of comparable size to those contained in hybrid polymersomes (Figure 4C). After endocytosis, hybrid polymersomes are transported through a trafficking vesicle to the endosome, where the pH is below the p*K*_a_ of the polymer. Inside the endosome, the PDPA block gets protonated and drives the disassembly of the hybrid polymersome. Nevertheless, gold nanoparticles grown *in situ* are expected to sequestrate some of the tertiary amino group labile to protonation and act as physical cross-linkers for the membrane,^29^ de facto preventing the complete disassembly of the hybrid nanostructure. We reproduced the endosomal conditions (*T* = 37 °C, pH 6) in hybrid polymersome dispersions and observed indicators of degradation by DLS, such as the overall reduction in the size of the assemblies, accompanied by the formation of larger unstable aggregates (Figure S16). The lower dispersity and the larger size of hybrid assemblies compared to those produced by pristine polymersomes in equivalent conditions (Figure S16B) suggest an incomplete disassembly process. The hybrid nanoassemblies encountered within the endosomes during TEM analysis conducted on T98G cells after incubation with hybrid polymersomes (Figure 4D) corroborate the DLS findings. The absence of an osmotic shock, which is achieved upon complete polymersome collapse,^54,55^ and the relatively large sizes of the observed assemblies are not compatible with their endosomal escape. Therefore, we hypothesize that the accumulation of hybrid assemblies within the endosome and the resulting proximity conditions are functional for reaching the thermal collective effects responsible for cell hyperthermal death.

We performed plasmonic hyperthermia experiments on T98G cells with two hybrid polymersome formulations. We incubated T98G cells with a dose of hybrid polymersomes corresponding to a gold concentration [Au] = 0.4 ppm (or *n*_AuNP_ ≈ 5 μm^–3^), at which more than 90% of the cells remained viable after 90 min of incubation (Figure S17). To excite the LSPR and induce hyperthermia, we applied a 514 nm laser using a scanning confocal microscope. The raster pattern of this light source provides a cumulative exposure time of only 3 min during the total 90 min of treatment. We monitored the real-time cell mortality via a live/dead fluorescence imaging assay (Movie S2) using two imaging lasers, 488 and 562 nm, for the excitation of the fluorophores labeling the live and dead cells, respectively. The intensity of both the imaging lasers was set to a 50-fold lower value than the LSPR excitation laser to minimize crosstalk and stray plasmonic stimulation.

For reference, we conducted negative control experiments monitoring the real-time mortality in the absence of LSPR excitation (“laser OFF” condition, Movie S3). Control cells treated with an equivalent dose of pristine polymersomes present only very sporadic and isolated dead cells after laser exposure (Movie S4), indicating that the laser irradiation alone was not noticeably phototoxic within the experimental time frame. Representative microscopy images taken before and after laser exposure are presented in the first and second columns of Figure 4B, respectively. In the cultures treated with hybrid polymersomes, the number of detected dead cells is remarkably higher after laser irradiation (top row) than in the laser OFF condition (bottom row). The column chart in Figure 4C provides a quantitative and comprehensive analysis of the mortality at the end of the *in vitro* thermoplasmonic exposure, as quantified by cell counting on live/dead stained T98G. In cells treated with hybrid polymersomes, the cell death rate depends on the laser intensity to which they were exposed, with mortalities that are maximum at 89% for *I* = 0.8 mW μm^–2^, decrease to 67% for *I* = 0.6 mW μm^–2^, and drop to only 13% when the laser stimulus is removed. As the differences between these values are statistically significant, we can infer that the mortality rate depends on the laser intensity, as does the temperature increase in our hybrid polymersomes (Figure S5C). The residual mortality recorded in the laser OFF case is ascribable to a combination of the nonzero intrinsic cytotoxicity of the internalized hybrid assemblies and their stray plasmonic excitation by the imaging laser lines. In contrast, the mortality of the cells treated with pristine polymersomes was negligible at 2–4% and independent of the laser intensity, thus confirming the absence of nonplasmonic phototoxicity. By analyzing the evolution of cell morphology during treatment (Figure S18), we detected features compatible with multiple or mixed cell death pathways. While a few cells presented blebbing and apoptotic bodies, we observed a widespread incidence of membrane rupture and extracellular release of intracellular material conventionally associated with primary or secondary necrotic death.^56,57^ Such variability may indicate spatial differences in the distribution of the uptaken hybrid assemblies and in the resulting local thermoplasmonic heating. The overall rapid cell death rates observed, with nearly 90% mortality recorded after just 90 min of treatment, are typically associated with nonapoptotic inflammatory pathways. Further investigation will be required to assign one or more specific pathways and determine whether an immune response is activated.

According to our proposed mechanism, the observed levels of laser-stimulated cytotoxicity are enabled by the accumulation of gold assemblies via receptor-mediated intracellular delivery. To experimentally support this argument, we repeated the *in vitro* hyperthermia treatment using nontargeting citrate-capped gold nanoparticles (cit-AuNPs). As shown in Figure S19, despite the double concentration needed to observe any cell death event, cit-AuNPs induced an almost 10 times lower mortality than hybrid polymersomes, with only 8.6% of the illuminated cells dying during the exposure. Such differences in photothermal effects must derive from a different final accumulation profile of gold caused by the dissimilar interactions of the two delivery systems with the cells.

Some factors should be considered to evaluate whether the observed cell death aligns with the uptake-enhanced thermoplasmonic response of our hybrid assemblies. Our measurements indicate that each cell internalizes over 25,000 hybrid polymersomes. Assuming a cell volume of 3500 μm^3^ and a homogeneous distribution, the average interassembly distance is less than 275 nm.

Our model predicts that under these conditions, the tested formulation can convert more than 52% of the incident laser power into heat, corresponding to approximately 0.26 mW. Using a simplified adiabatic approximation, this energy input is sufficient to locally raise intracellular temperatures well above the 43 °C required to initiate hyperthermal death^58,59^ within milliseconds of laser exposure. While such simplified speculations cannot precisely predict the temperature reached within the crowded and heterogeneous intracellular environment, they do suggest that the expected order of magnitude is consistent with the observed effects.

To further investigate the causes of cell death, we examined the potential role of concurrent photochemical effects. These are defined as the generation of reactive oxygen species (ROS) at cytotoxic concentrations as a result of the direct charge or energy transfer from laser-activated gold nanoparticles to molecular oxygen.^60,61^ We treated T98G cells with either hybrid or pristine polymersomes under laser ON and OFF conditions and labeled them with a generic ROS probe. Fluorimetry data showed no significant changes in ROS concentrations compared to the basal level measured in untreated cells under any tested condition (Figure S20). Although ROS can also be endogenously generated by thermal distress,^62^ the rapid onset of cell death in our cultures, with complete loss of membrane integrity within 90 min, suggests that the time frame is too short for metabolic ROS production to be detectable. Instead, our findings align with direct photothermal disruption as the predominant origin of cell death.

## Conclusions

In conclusion, our research reveals that the *in situ* synthesis of gold nanoparticles within polymersome membranes provides a simple yet powerful approach for generating a thermoplasmonic system. Our reaction operates in aqueous environments under mild conditions and does not require sophisticated equipment or controlled atmospheres, offering potential for easy scale-up. Precise control over gold nanoparticle size and loading is achieved by merely modifying the reaction stoichiometry. Reproducibility is demonstrated through 24 formulations from five polymersome batches. Notably, the resulting hybrid polymersomes exhibit exceptional colloidal stability, maintaining their integrity for over a year. By optimizing our formulation, we enhanced the thermoplasmonic performance of these systems, achieving temperature increases exceeding 10 K in dispersion. These results conclusively demonstrate that hybrid polymersomes containing *in situ*-grown small gold nanoparticles can serve as efficient collective plasmonic heating solutions when in close range. We also developed a theoretical model that aligns closely with our experimental data, offering a deeper understanding of the collective nature of the photothermal mechanism. This novel theoretical framework integrates a semiclassical approach to quantum confinement effects in small plasmonic nanoparticles with the optical contribution of polymer scaffolds, offering a precise description of the thermoplasmonic response of hybrid assemblies. Furthermore, it effectively accounts for heat dissipation in the experimental setup, enabling seamless adaptation to application-specific environments. Overall, the strong agreement between our theory and experiments establishes a robust foundation for the future refinement and design of light-activated nanoreactors with precise temperature control using hybrid systems.

Finally, our proof-of-concept *in vitro* experiments on T98G human glioblastoma cells demonstrate the biomedical potential of these polymersomes. Their photothermal cytotoxicity, which resulted in up to 90% cell death after brief laser exposure, showcases their effectiveness as light-activated intracellular heaters. While we acknowledge that endogenous chromophores pose challenges for plasmonic hyperthermia in the visible spectrum, these limitations can be mitigated by targeting superficial applications or utilizing catheter-assisted light delivery during surgical procedures.^49^ Although we tailored the laser stimuli to align with the resonance of our gold nanoparticles in this study, future research can adapt our methodology to near-infrared (NIR)-compatible particles or integrate it with upconverting nanomaterials^49,63^ to enhance tissue penetration. The modeling framework and supramolecular design strategies outlined here are broadly applicable, underscoring the potential of hybrid polymersomes for biomedical applications that require microscale temperature control despite current wavelength limitations.

## Materials and Methods

### Materials

Tetrahydrofuran (THF, 99.8% HPLC grade) and methanol (MeOH, 99.9% HPLC grade) were purchased from Thermo Scientific Chemicals. For the phosphate buffer (PB), sodium phosphate monobasic (≥99.0%) and sodium phosphate dibasic (≥99.0%) were purchased from Sigma-Aldrich. Milli-Q water (18.2 MΩ·cm, <5 ppb total organic carbon species) was produced through a Milli-Q Reference ultrapure water system equipped with a BioPak end filter (Merck Millipore). Phosphate buffer saline (PBS, calcium- and magnesium-free) tablets were purchased from Gibco and dissolved in Milli-Q water according to the supplier’s instructions to obtain PBS 1× pH 7.4 buffer. Gold(III) chloride trihydrate (HAuCl4:3H2O, ≥99.9% trace metals basis) and sodium borohydride (NaBH4, 99.99% trace metals basis) were purchased from Sigma-Aldrich. Concentrated nitric (67–69% in water, trace metal, for trace metal analysis) and hydrochloric (37% in water, extra pure) acids were purchased from Fisher Chemicals. The specifications and suppliers of other protocol-specific reagents and instruments are provided within the respective dedicated subsections.

### Self-Assembly of PMPC–PDPA Polymersomes

The PMPC_20_–PDPA_80_ copolymer was synthesized by atom transfer radical polymerization (ATRP) using a previously published protocol.^27,64^ PMPC–PDPA polymersomes were fabricated by the bottom-up solvent switch method. In a typical experiment, 20 mg of PMPC_20_–PDPA_80_ was dissolved in 1 mL of THF/MeOH (1:3). After complete dissolution, 2.3 mL of aqueous buffer (water, phosphate buffer 0.1 M, or PBS 1×) was injected via an automatic syringe pump at 2.0 μL min^–1^ into the polymer solution under stirring at 40 °C. The reaction was quenched upon completion by adding 2.7 mL of the aqueous buffer solution at once and dialyzed against PBS 1× overnight with a 3.5 kDa molecular cutoff membrane (Repligen). The polymersome dispersions were centrifuged for 10 min at 21 °C and 1000 relative centrifugal force (RCF) units to eliminate large polymer aggregates and then further purified by size exclusion chromatography through a column packed with agarose gel (Sepharose 4B, Sigma-Aldrich). The dispersions were concentrated by tangential flow filtration through a hollow fiber module with 100 kDa MWCO (Repligen) when needed.

### Synthesis of Hybrid Polymersomes

Adequate volumes of a 50 mM HAuCl_4_:3H_2_O stock solution to reach the desired Au/DPA mol % were added dropwise to a PMPC–PDPA polymersome dispersion under vigorous stirring in an ice bath. The sample was kept stirring under pH monitoring to allow the partial protonation of the PDPA blocks and the diffusion of the AuCl_4_^–^ ions to the membrane. After 30 min, an equivalent volume of freshly prepared ice-cold 50 mM NaBH_4_ solution was added to the sample at once. Samples were immediately transferred to a 3.5 kDa MWCO dialysis membrane and dialyzed against PBS 1× pH 7.4 to re-equilibrate the pH and remove unreacted species. To eliminate gold nanoparticles that may nucleate in the solvent after membrane saturation, the hybrid polymersome dispersions were purified by repeated cycles of tangential flow filtration through a hollow fiber module with 0.05 μm cutoff (Repligen) until generating a filtrate of 40x the sample volume.

### Morphological Characterization of Pristine and Hybrid Polymersomes

The size distribution of pristine and hybrid polymersomes was determined by dynamic light scattering (DLS) on a Zetasizer Nano ZS (Malvern Panalytical) instrument equipped with a 120 mW 630 nm He–Ne laser in a 173° “backscattering” detector configuration at a controlled temperature of 25 °C. Samples were diluted to 0.1 g L^–1^ with PBS 1× before the measurement. The morphology of the assembled structures generated by the different prepared formulations was determined by transmission electron microscopy (TEM). TEM specimens were prepared by drop-casting 5 μL of sample onto a carbon-coated Cu grid, previously glow-discharged for 45 s. After 1 min, the excess sample was drained from the grid by blotting with filter paper. A 0.5 wt % PTA positive staining solution was prepared by dissolving phosphotungstate octadecahydrate (2Na2O·P2O5·12WO3·18H2O, Sigma-Aldrich) in Milli-Q water. The staining was applied by immersing the specimen grid for 3 s in a 20 μL drop of 0.5 wt % PTA. The excess staining solution was drained again, and the grid was further dried under vacuum for 1 min. The grids were imaged at 200 kV using a JEOL JEM-2100 TEM equipped with a Gatan Orius SC-200 camera.

### Concentration Measurements

The final polymer concentration was determined by reverse-phase high-performance liquid chromatography (RP-HPLC) using a Jupiter C18 column (Phenomenex). Before the analysis, an aliquot of each sample was dissolved into a 24:1 PBS pH 1/NaCl 1 M acid mixture to solubilize the polymer and centrifuged three times at 20,000 RCF for 1 h at room temperature to precipitate the gold nanoparticles. The supernatant was passed through the HPLC column using a mixture of methanol/water in a linear concentration gradient as a mobile phase injected at 1 mL min^–1^ to allow isolation of the polymer component. The polymer concentration was quantified from the UV absorption at 220 nm.

The Au concentration was measured by microwave-assisted plasma atomic emission spectroscopy (MP-AES). An aliquot of each sample was dissolved in a 2× volume of aqua regia (3:1 HCl/HNO_3_), sonicated for 45 min, and left overnight to allow for the complete solvation of the Au ions. The solutions were then diluted 10:1 with Milli-Q water and analyzed with an Agilent 4100 MP-AES instrument. Au was detected by the emission lines at 242.795 and 267.595 nm, and the concentration was determined from the instrument’s calibration with an ICP grade Au calibration standard (5190, Agilent).

### UV–Vis Absorption Spectroscopy

UV–vis extinction spectra of pristine and hybrid polymersomes were collected with a Shimadzu UV-2700 UV–vis spectrophotometer using quartz cells with a light path length *l* = 10 mm and PBS 1× as a reference. The extinction value was indirectly calculated as *E* = −log_10_(Φ_t_/Φ_0_), where Φ_t_ and Φ_0_ are the transmitted and incident photon fluxes, respectively. Absorption spectra of hybrid polymersomes were calculated by subtraction of the extinction spectra of pristine polymersomes at the same concentration under the assumption of negligible absorption of polymersomes and negligible scattering of gold nanoparticles in the visible range.

### Temperature Measurements

In a typical experiment, 300 μL of an aqueous dispersion of hybrid or pristine polymersomes was transferred to a 10 mm path length quartz cuvette and exposed to a solid-state continuum 532 nm laser beam (Gem 532, Laser Quantum) focused through a plano-convex lens with focal length *f* = 50 mm (LA1131-A-ML, Thorlabs). Where not specified, the nominal laser power was set to 200 mW, corresponding to an incident power on the cuvette of 180 mW. Meanwhile, the evolution of temperature was monitored through an in-house resistance temperature detector (RTD) composed of a 4-wire PT-100 probe and a MAX31865 analog-to-digital (RS Components) mounted onto an Arduino Uno board, offering a sensitivity of ±0.03 K. The data acquisition Arduino library “Adafruit_MAX31865” was developed under a BSD license by Limor Fried/Ladyada at Adafruit Industries. For selected samples, the spatial evolution of the temperature upon irradiation in the cuvette was imaged through a FLIR A6703 IR camera (f/4.0 aperture and 60 Hz frame rate) positioned at a 90° angle with respect to the laser beam path.

### Optical Calculations

The extinction cross sections of pristine and hybrid polymersomes were calculated using a Mie theory approach. The polymersome was modeled as a four-layer stratified spherical particle (Figure S12A) composed of a water core surrounded by three polymer layers: a hydrophobic PDPA layer (containing the gold nanoparticles in the hybrid case) sandwiched between two hydrated PMPC ones. The thicknesses of the three layers were evaluated by semiempirical scaling laws of the form *bM*^υ^, where *b* is the monomer length, *M* the degree of polymerization, and υ is the Flory exponent, υ = 0.9 for PMPC and υ = 2/3 for PDPA.^40^ The Mie calculations were carried out using an adaptation of the code contained in the MatScat MATLAB package.^65−68^ The hydrophobic PDPA layer was optically modeled as poly(methyl methacrylate) (PMMA), while the refractive index of the hydrated PMCP layers was calculated by the Bruggeman effective medium approximation^69^ using dry PMPC^70^ and water as boundary media. The water volume fraction ϕ_w_ = 0.39 was estimated from experimental data on bound water in PMPC brushes reported by Hatakeyama et al.^71^ The resulting effective refractive index is 1.43. The size-dependent complex permittivities of ultrasmall gold nanoparticles were calculated by extrapolation of the semiempirical model by Karimi et al.^45^ The absorption cross section was determined in the quasi-static approximation of the Mie theory as , where λ_0_ is the incident wavelength and ε(*a*) and ε_m_ are the electric permittivities of the gold nanoparticle of radius *a* and of PMMA, respectively.

### Thermoplasmonic Simulations

The light–matter interaction of isolated hybrid polymersomes was numerically simulated by means of finite element methods (FEM), in particular using COMSOL Multiphysics 5.6, a software offering integrated solid methods for the solution of partial differential problems on complex geometries. The geometry and materials composing the system were assigned according to the stratified sphere model (Figure S12A) described in the Optical Calculations section. The effective thermal conductivity of the hydrated PMPC layers *k* = 0.292 W m^–1^ K^–1^ was estimated by applying the Bruggeman effective medium approximation^69^ with PMMA and water as boundary media at a water volume fraction ϕ_w_ = 0.39. A randomly distributed array of χ_AuNP_ gold nanoparticles with radius *a* = 1 nm was embedded within the membrane. All structures were meshed with a wavelength-controlled free tetrahedral mesh having a maximum size of 0.33 nm for the gold nanoparticles and 1 nm for the polymer layers (Figure S12B). A combination of scattering boundary limits (set to 12× the structure size) and perfectly matched layer (PML) domains were used as boundary conditions to avoid computational artifacts coming from parasite optical events, e.g., artificial reflections. The simulation workflow entails two distinct steps. The first is the electromagnetic part of the problem, which was solved using the RF Module under illumination by a linearly polarized plane wave of wavelength λ = 532 nm and intensity *I* = 0.128 mW μm^–2^. The second step is heat transfer, which was solved by applying the heat transfer in solids (HTS) interface to find the solution of the heat equation considering the resistive losses calculated in the previous step as a heat source and a heat flux node (convective interface) along the outer boundaries as a heat sink.

### T98G Cell Culture

The T98G human glioblastoma cells were kindly provided by Prof. Vivaldo Moura Neto (IECPN, Brazil). This cell line originated from a white, 61-year-old male and presents a fibroblast-like cell phenotype. Cells were cultured in Dulbecco’s modified Eagle Medium-GlutaMAX with glucose and sodium pyruvate (10569010, Gibco), supplemented with 10% fetal bovine serum (FBS, A5670701, Gibco) and 1% penicillin/streptomycin mixture (10,000 U/mL, 15140122, Gibco) at 37 °C in a 5% CO_2_ atmosphere. For subcultures, after reaching confluency, the cells were detached from the flasks, using 0.25% (w/v) trypsin/EDTA (25200056, Gibco) for 5 min at 37 °C and centrifuged at 200 RCF for 5 min. Different cellular densities were used according to each experiment.

### MTT Metabolic Assay

T98G cells were cultured into 96-well plates at a density of 10^4^ cells per well, overnight at 37 °C in a 5% CO_2_ atmosphere. Then, cell cultures within the same row were incubated with serial 2× dilutions of hybrid or pristinepolymersome dispersion (starting polymer concentration *c* = 0.1 g L^–1^) for 1.5 h. Control cultures were grown on the same plate and maintained in culture medium. Thereafter, cell viability was assessed by the MTT reduction colorimetric assay by adding 50 μL of MTT reagent (M6494, Molecular Probes) to each well (0.5 g L^–1^). After 2 h, the production of water-insoluble purple formazan crystals in metabolically active cells was observed, which were then dissolved by adding 100 μL of DMSO (D8418, Sigma-Aldrich). The absorbance of solubilized formazan at 570 nm was measured in a plate reader and normalized to that of control wells.

### Western Blotting

To generate T98G cell lysates, 10^6^ cells were washed with PBS and centrifuged at 400 RCF twice. Cell pellets were then lysed with 100 μL of RIPA buffer (R0278, Sigma-Aldrich) including protease inhibitors (1:100, P8340, Sigma-Aldrich) for 30 min at 4 °C. Cell lysates were further centrifuged at 17,000 RCF, 4 °C for 20 min, after which supernatants were collected and protein content quantified by Bradford assay following the manufacturer’s protocol (#5000006 Protein Assay Dye Reagent, Bio-Rad). For Western blot analysis, proteins were denatured with 2× Laemmli buffer (1610737, Bio-Rad) and β-mercaptoethanol (1610710, Bio-Rad) at 95 °C for 5 min prior to sample addition into 12% Bis–Tris acrylamide gel (TGX FastCast Acrylamide Kit, #1610175) containing 10% sodium dodecyl sulfate (BioUltra SDS, 71736, Sigma-Aldrich). A total of 20 μg of protein (40 μL maximum per well) was added to gels, on which electrophoresis was run for 30 min at 80 V, followed by 1.5 h at 120 V using a Bio-Rad Power Pac source system in 1× Tris–Glycine running buffer (1610735, Bio-Rad). After electrophoresis, proteins were transferred into polyvinylidene difluoride membranes (Immun-Blot 1620174, Bio-Rad) using a wet transfer system in Tris–Glycine buffer with 20% methanol at 50 V for 1 h, followed by 100 V for 1 h at 4 °C. Subsequently, the membrane was blocked with 5% nonfat dried milk powder (PanReac, AppliChem) in Tris–Buffer Saline (TBS, 1706435, Bio-Rad) for 1 h at room temperature. Membranes were then incubated with diluted primary antibodies to an adequate working concentration in a solution of 1% milk in TBS+0.1%Tween (TBS-T, Tween 20 from Sigma-Aldrich) at 4 °C for detecting the specific scavenger receptors: anti-SR-B1 from rabbit (Novus Biologicals, NB400-131SS, dilution 1:1000) and anti-CD36 from rabbit (Novus Biological NB400-144SS, dilution 1:500). Membranes were further probed for glyceraldehyde 3-phosphate dehydrogenase, using a-GAPDH from mouse (Proteintech, 60004-1-Ig, dilution 1:10,000) as a loading control. Next, the membranes were washed three times with TBS-T, and the corresponding diluted secondary antibodies were incubated for 1 h at room temperature and washed again three times with TBS-T. Membranes were imaged on a Licor CXF system (Odyssey) and analyzed in the Fiji image analysis software.

### Au Cellular Uptake

T98G cells were plated in tissue-culture-treated 6-well plates at a density of 5 × 10^5^ cells per well and grown for 24 h. In each well, the cells were incubated with 1 mL of FBS-supplemented culture medium containing ≈4.5 × 10^11^ hybrid polymersomes (total Au mass: 6.4 μg) for 1 h at 37 °C and 5% CO_2_. In control experiments, cells were treated with comparable doses of pristine PMPC–PDPA polymersomes or commercial 1.8 nm citrate-capped gold nanoparticles (A11-1.8-CIT-PBS, Nanopartz) dispersed in the FBS-supplemented medium. After incubation, the cells were washed three times with PBS 1× to eliminate the excess nanoparticles and detached by incubation with 1 mL of trypsin/EDTA 0.25% (25200056, Gibco) for 5 min. After centrifugation at 200 RCF for 5 min, the pellets were redispersed in 1 mL of fresh culture medium for counting with a TC20 automated cell counter (Bio-Rad). After reprecipitation, the obtained pellets were treated with 150 μL of aqua regia (3:1 HCl/HNO_3_), sonicated for 45 min at room temperature, and left overnight to allow for the complete digestion of the organic matrix and solvation of the Au ions. The solutions were then diluted 10:1 with Milli-Q water, and the concentration of Au was measured with an Agilent 4100 MP-AES instrument (Au emission line at 267.595 nm) after calibration of the instrument with an ICP grade Au calibration standard (5190, Agilent).

### TEM Imaging of Cells

T98G cells were incubated with hybrid polymersomes for 1 h following the protocol described in the Au Cellular Uptake Section. After the second round of centrifugation, cell pellets were fixed with 2.5% glutaraldehyde (Sigma-Aldrich) and 2% paraformaldehyde (Sigma-Aldrich) in 0.1 M phosphate buffer pH 7.4. Samples were postfixed with osmium tetroxide and dehydrated with acetone, embedded in Spurr epoxy resin, and sectioned using Leica ultramicrotome UC7 (Leica Microsystems). Ultrathin sections (≈60 nm) were analyzed with a JEOL JEM-1010 TEM fitted with an Orius SC1000 (model 832, Gatan) digital camera. All the specimen preparation and imaging steps were carried out by the TEM-SEM Electron Microscopy Unit, Scientific and Technological Centers of the University of Barcelona (CCiTUB).

### In Vitro Plasmonic Hyperthermia Experiments

Initially, 1.2 × 10^4^ T98G cells per well were plated on tissue-culture-treated microscopy grade eight-well plates (80826, ibidi) and grown for 24 h. The live/dead fluorescence labeling kit (R37601, Invitrogen) was prepared by dissolving the lyophilized “dead” label, BOBO-3 iodide, into the “live” staining calcein acetoxymethyl solution. A dose of 100 nm hybrid polymersomes to give a final [Au] = 0.4 ppm was dispersed in 100 μL of live cell imaging solution (A59688DJ, Invitrogen) and mixed with 100 μL of freshly prepared live/dead staining solution. The cell culture medium was removed from the well, and the cells were incubated with the hybrid polymersome/staining solution for 15 min at 37 °C, 5% CO_2_. Control experiments were carried out using an equivalent dose of pristine polymersomes (0.01 g L^–1^ of polymer) or a double Au dose, [Au] = 0.8 ppm, of 1.8 nm citrate-capped gold nanoparticles (A11-1.8-CIT-PBS, Nanopartz). Incubated cells were handled avoiding exposure to stray lights. After incubation, the cells were transferred to the environmental chamber of a Leica TCS SP8 confocal microscope, where laser stimulation and real-time fluorescence imaging were carried out in alternated cycles. The laser stimulation cycle was performed by focusing the 514 nm line of an Ar^+^ laser at 0.5 and 0.35 mW through a 20× objective (NA = 0.70) on the cells and raster scanning it across the field of view with a pixel dwell time of 1.2 μs. During the imaging cycle, the “live” and “dead” fluorescent labels were excited using the 488 nm line of an Ar^+^ laser and the 561 nm line of a diode-pumped solid-state laser, respectively. Bright-field images were also collected, illuminating the cells with a 405 nm diode laser. To minimize photodegradation and avoid stray thermoplasmonic stimulation during this cycle, the power of imaging lasers was kept at *P* ≤ 0.01 mW. The two cycles were alternated for 90 min with a period of 9.25 s, leading to a total 514 nm-laser exposure time of ∼3 min. Negative control experiments were carried out by keeping the 514 nm laser line off during the stimulation cycle.

### Reactive Oxygen Species (ROS) Evolution Assay

T98G cells were plated at a density of 10^4^ cells per well in black 96-well flat-bottomed plates and grown overnight. The culture medium was replaced with fresh culture medium containing a dose of hybrid polymersomes to give [Au] = 0.4 ppm. In control experiments, the cells were treated with an equivalent dose of pristine polymersomes. The cells were incubated in the environmental chamber (37 °C, 5% CO_2_) of a Leica TCS SP8 confocal microscope, and the central area of the well was exposed to a 514 nm laser line in the same scanning illumination conditions described in the previous section (0.5 mW and laser off conditions). After the treatment, 100 μL of Cellular ROS Deep Red staining solution (ab186029, Abcam) was added to the cells, which were then incubated for 45 min at 37 °C, 5% CO_2_. Untreated T98G cells were stained with the same procedure for reference. The ROS-activated fluorescence signal was detected with a Tecan Spark fluorimeter in top reading mode in the center of the well with an excitation wavelength of 650 ± 5 nm and an emission wavelength of 675 ± 5 nm. Each measure was collected over 30 flashes of 40 μs each. The optimal *z*-position was determined as the height at which the maximal fluorescence signal was detected during a preliminary *z*-scan.

### Statistical Analysis

To accurately determine statistical descriptors, the asymmetrical size distributions of both polymersomes and gold nanoparticles were normalized by a Box–Cox transformation in MATLAB. In the case of relative distributions measured by DLS, corresponding 1000-particle populations were generated by a resampling-with-replacement approach prior to transformation.^72^ After transformation, data points lying outside the region defined as 1.5 times the interquintile range were excluded as outliers. Transformed and cleaned populations passed the Jarque–Brera test with a significance level α = 0.05. The means and confidence intervals of the original distributions were calculated by back-transforming the means and standard deviations of the normalized distributions. The statistical significance of all the presented biological assays was determined by applying suitable tests (each indicated in the corresponding figure caption) within the GraphPad Prism software package.

## Data Availability

The datasets supporting this study are available in the repository at https://doi.org/10.6084/m9.figshare.28546346.v1,^73^ under a CC-BY 4.0 licence.

## Supporting Information Available

Detailed calculations for gold loading estimation; full derivation of the theoretical heat generation and transfer model in hybrid polymersome dispersions; additional morphological, compositional, and physicochemical characterization of hybrid polymersomes; experimental measurements and theoretical modeling of the hybrid polymersomes’ thermoplasmonic response; and supporting assays to the application of hybrid polymersomes as intracellular heaters in *in vitro* plasmonic hyperthermia experiments (PDF)
Thermoplasmonic heating of hybrid polymersome dispersions, captured via thermal imaging (AVI)
Real-time mortality of T98G cells treated with hybrid polymersomes under laser exposure via a live/dead fluorescence imaging assay (AVI)
Live/dead fluorescence imaging assay of T98G treated with hybrid polymersomes in the “Laser OFF” condition (AVI)
Live/dead fluorescence imaging assay of control cells treated with an equivalent dose of pristine polymersomes presenting only very sporadic and isolated dead cells after laser exposure (AVI)
Source data (XLSX)

## Supplementary Material

nn4c14093_si_001.pdf

nn4c14093_si_002.avi

nn4c14093_si_003.avi

nn4c14093_si_004.avi

nn4c14093_si_005.avi
