# Supplementary material for: Thermoplasmonic Polymersome Membranes by In Situ Synthesis
Source: ACS Nano. 2025 Apr 18;19(16):15331–44. doi: 10.1021/acsnano.4c14093 (PMC12045020; doi:10.1021/acsnano.4c14093)
Supplement: Supplementary file 1 — nn4c14093_si_001.pdf [file nn4c14093_si_001.pdf]

Supporting Information for

Thermoplasmonic polymersome membranes by *in situ* synthesis

Valentino Barbieri[1,2], Javier González Colsa[3], Diana Matias[1,2,4], Aroa Duro Castano[1,†], Anshu Thapa[1], Lorena Ruiz-Pérez[1,2,5], Pablo Albella[3], Giorgio Volpe[1,] and Giuseppe Battaglia[1,2,6,*]*

[1] Department of Chemistry, University College London, WC1H 0AJ, London, United Kingdom.

[2] Institute for Bioengineering of Catalunya (IBEC), The Barcelona Institute of Science and Technology (BIST), 08028, Barcelona, Spain.

[3] Group of Optics, Department of Applied Physics, University of Cantabria, 39005, Santander, Spain.

[4] Instituto de Medicina Molecular João Lobo Antunes (iMM), 1649-028, Lisbon, Portugal.

[5] Serra Húnter Fellow, Department of Applied Physics, University of Barcelona, 08028, Barcelona, Spain.

[6] Catalan Institution for Research and Advanced Studies (ICREA), 08010, Barcelona, Spain.

*Corresponding authors' emails:
Giorgio Volpe: g.volpe@ucl.ac.uk
Giuseppe Battaglia: gbattaglia@ibecbarcelona.eu

SUPPLEMENTARY TEXT

Au Loading Estimation

The number density of polymersomes n and the total membrane volume V_m were determined by combining concentration data and DLS size distributions. The number density of polymersomes having radius R_i in the discrete size distribution $f_i = f(R_i)$ is:

$$n_i = \frac{N_i}{N_{\mathrm{agg},i}}, \tag{S1}$$

with N_i being the total number of polymer molecules in the bin i and $N_{\mathrm{agg},i}$ is the aggregation number, i.e., the number of polymer molecules in a polymersome calculated as:

$$N_{\mathrm{agg},i} = \frac{pV_{\mathrm{m},i}}{v}, \tag{S2}$$

where v is the molecular volume of the PDPA block, p is the packing parameter ($p \sim 1$ for vesicular systems) and $\mathrm{V}_{\mathrm{m},i} = \frac{4}{3}\pi(R_i - b)^3 - (R_i - b - m)^3$ is the membrane volume of the polymersome of radius R_i having brush and membrane thicknesses b and m, respectively. N_i can be calculated as:

$$N_i = cN_\mathrm{A} \cdot \frac{f_i N_{\mathrm{agg},i}}{\sum_i f_i N_{\mathrm{agg},i}}, \tag{S3}$$

where c is the molar concentration of polymer measured by RP-HPLC and N_A the Avogadro's constant. Therefore, inserting (S3) in (S1) we obtain:

$$n_i = cN_\mathrm{A} \cdot \frac{f_i}{\sum_i f_i N_{\mathrm{agg},i}}. \tag{S4}$$

A weighted sum of all n_i over the whole size distribution gives the total number density:

$$n = \sum_{\text{i}} n_i \,. \tag{S5}$$

At this point, the total volume fraction of PDPA membranes in the sample can be obtained as:

$$\phi_{\text{m}} = \sum_{i} n_i V_{\text{m},i} \,. \tag{S6}$$

The total volume fraction of gold in the sample is calculated as:

$$\phi_{\text{Au}} = \frac{c_{A\text{u}}}{10^6 \cdot \rho_{\text{Au}}} \tag{S7}$$

where c_{Au} is the concentration of Au in ppm calculated by MP-AES and ρ_{Au} the density of Au in g cm^{-3}. The total number density n_{AuNP} [cm^{-3}] can then be obtained as:

$$n_{\text{AuNP}} = \frac{\phi_{\text{Au}}}{V_{\text{AuNP}}}, \tag{S8}$$

where $V_{\text{AuNP}} = \frac{4}{3}\pi a^3$ is the volume of a spherical particle of radius a.

Finally, the two loading indicators, i.e., the loading volume ratio $\varphi_{\text{Au/m}}$ and the loading capacity per polymersome χ_{AuNP} are calculated as follows:

$$\varphi_{\text{Au/m}} = \frac{\phi_{\text{Au}}}{\phi_{\text{m}}}, \qquad \chi_{\text{AuNP}} = \frac{n_{\text{AuNP}}}{n}. \tag{S9}$$

Theoretical model of the heat generation and transfer in hybrid polymersome dispersions

A model was elaborated to describe the evolution of the observed temperature increment ΔT upon laser illumination (Figure 3).

The following hypotheses were applied:

1. Gold nanoparticles are much smaller than the incident wavelength (quasi-static approximation).
2. The polymersome absorption and gold nanoparticle scattering are negligible in the visible range.
3. No electromagnetic coupling occurs between gold nanoparticles, which are considered independent dipoles.
4. The concentration is in the validity range of Beer-Lambert's law.
5. The incident beam has a Gaussian power distribution.
6. The concentration of the hybrid polymersomes is homogenous in the sample.

Under hypotheses 1 and 2, the extinction cross-section of a single hybrid polymersome, σ_{ext}, can be expressed as a linear combination of the scattering cross-section of the polymer shell (σ_{sca}) and the absorption cross-sections (σ_{abs}) of all the χ_{AuNP} embedded gold nanoparticles (see previous section): $\sigma_{\text{ext}} = \chi_{\text{AuNP}}\, \sigma_{\text{abs}} + \sigma_{\text{sca}}$.

Scattering and absorption events attenuate the intensity I of the laser beam as it proceeds through the sample, so that $I = I(\vec{r}, n)$, where $\vec{r} = (x, y, z)$ is the spatial coordinate of a generic point in the illuminated volume and n is the number density of hybrid polymersomes.

Upon laser illumination, the LSPR also results in the generation of thermoplasmonic heating. The total generated heat flow rate is given by:[1]

$$\dot{Q}(\vec{r}, n) = \sigma_{\text{abs}}\, I(\vec{r}, n)\, N_{\text{AuNP}}, \tag{S10}$$

where N_{AuNP} represents the total number of gold nanoparticles acting as absorbers/emitters, i.e., particles within the illuminated volume V.

Given the homogeneous random distribution across the whole dispersion (hypothesis 6), we can assume that $N_{\text{AuNP}} = n_{\text{AuNP}} V$ with n_{AuNP} being the number density of gold nanoparticles, which in turn can be related to the hybrid polymersomes number density, n, through: $n_{\text{AuNP}} = n\, \chi_{\text{AuNP}}$.

Therefore, in the infinitesimal volume element $\mathrm{d}V$, an infinitesimal heat flow rate is produced:

$$\mathrm{d}\dot{Q}(\vec{r},n) = n\,\chi_{\mathrm{AuNP}}\,\sigma_{\mathrm{abs}}\,I(\vec{r},n)\,\mathrm{d}V. \tag{S11}$$

In the absence of the sample, the spatial dependence of the laser resides only in the Gaussian intensity profile (hypothesis 5):

$$I_0(x,y,z) = \frac{2P}{\pi[\omega(z)]^2}e^{-\frac{2(x^2+y^2)}{[\omega(z)]^2}}, \tag{S12}$$

where P is the laser power and the function $\omega(z)$ describes the Gaussian waist radius along z. It can be demonstrated that in free space at any z:

$$\iint_{-\infty}^{\infty} I_0(x,y,z)\,\mathrm{d}x\,\mathrm{d}y = P. \tag{S13}$$

As the power is conserved along z, when no optically active species are present, the incident intensity can be expressed as:

$$I_0(x,y,z) \approx I_0(z) = \frac{P}{\pi[\omega(z)]^2} = \frac{P}{A(z)}, \tag{S14}$$

with $A(z)$ being the cross-sectional area of the propagating beam. When the sample is present, attenuation occurs along the propagation direction z due to absorption and scattering according to the Beer-Lambert law (hypothesis 4):

$$I(n,z) = I_0(z)e^{-n\sigma_{\mathrm{ext}}z} = I_0(z)\,e^{-n(\chi_{\mathrm{AuNP}}\,\sigma_{\mathrm{abs}}+\sigma_{\mathrm{sca}})z}. \tag{S15}$$

By substituting Equation S14 into Equation S15:

$$I(n,z) = \frac{P}{A(z)}e^{-n\sigma_{\mathrm{ext}}z}. \tag{S16}$$

The laser intensity can be substituted into Equation S11, expressing the infinitesimal volume as a function of z, $\mathrm{d}V = A(z)\,\mathrm{d}z$. This substitution gives

$$\mathrm{d}\dot{Q}(n,z) = n\,\chi_{\mathrm{AuNP}}\,\sigma_{\mathrm{abs}}\,Pe^{-n\sigma_{\mathrm{ext}}z}\,\mathrm{d}z. \tag{S17}$$

Integration of $\mathrm{d}\dot{Q}(n,z)$ with respect to z from 0 to l gives:

$$\dot{Q}(n) = P\,\frac{\chi_{\mathrm{AuNP}}\;\sigma_{\mathrm{abs}}}{\sigma_{\mathrm{ext}}}\,(1 - e^{-n\sigma_{\mathrm{ext}}l}). \tag{S18}$$

The generated heat is exchanged with the environment through convection boundary conditions along the cuvette wall surfaces, then followed by conduction across the quartz walls. At the steady state, the following energy balance is reached:

$$\dot{Q}(n) = \dot{Q}_{\mathrm{conv}} + \dot{Q}_{\mathrm{cond}}. \tag{S19}$$

The terms on the right-hand side can be expanded as follows

$$\dot{Q}(n) = \Delta T\sum_i h_i A_i + \frac{kS}{L}\Delta T = \beta\Delta T, \tag{S20}$$

where h_i and A_i are the convective heat transfer coefficients and areas of the cooling surfaces. In particular h_i values were estimated from the Nusselt number correlations for vertical and horizontal planes.[2,3] k, S and L are the conductivity, transversal section, and length of the quartz cuvette wall in contact with the sample, respectively. These parameters can all be summed up in the generalized heat transfer coefficient β. The increment in temperature obtained at thermal equilibrium is therefore

$$\Delta T(n) = \frac{P}{\beta}\,\frac{\chi_{\mathrm{AuNP}}\;\sigma_{\mathrm{abs}}}{\sigma_{\mathrm{ext}}}\,(1 - e^{-n\sigma_{\mathrm{ext}}l}). \tag{S21}$$

By simple variable changes ΔT can also be expressed as a function of the mean inter-polymersome distance $\delta = \left(\frac{4\pi n}{3}\right)^{-1/3} \Gamma\left(\frac{4}{3}\right) = \kappa n^{-1/3}$,[4]

$$\Delta T(\delta) = \frac{P}{\beta} \frac{\chi_{\mathrm{AuNP}}\, \sigma_{\mathrm{abs}}}{\sigma_{\mathrm{ext}}} \left[1 - e^{-\sigma_{\mathrm{ext}} l \left(\frac{\kappa}{\delta}\right)^3}\right], \tag{S22}$$

or of the gold nanoparticle number density $n_{\mathrm{AuNP}} = n\, \chi_{\mathrm{AuNP}}$,

$$\Delta T(n_{\mathrm{AuNP}}) = \frac{P}{\beta} \frac{\chi_{\mathrm{AuNP}}\ \sigma_{\mathrm{abs}}}{\sigma_{\mathrm{ext}}} \left(1 - \mathrm{e}^{-\frac{\sigma_{\mathrm{ext}} l}{\chi_{\mathrm{AuNP}}} n_{\mathrm{AuNP}}}\right). \tag{S23}$$

SUPPLEMENTARY FIGURES

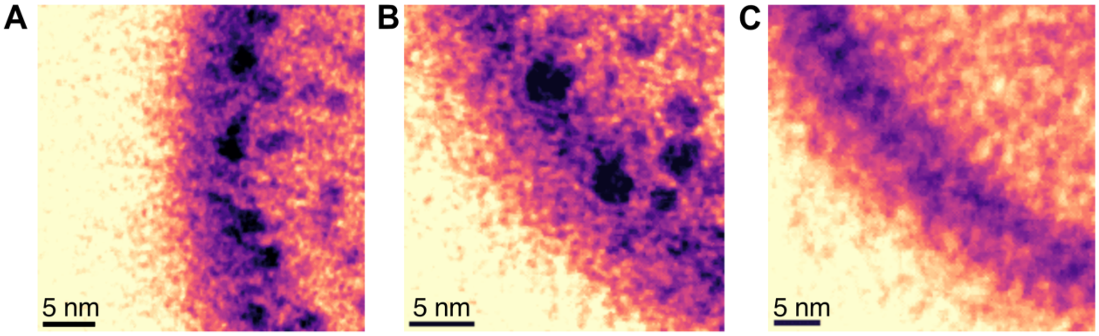

Figure S1. TEM analysis shows the complete embedment of gold nanoparticles and the PDPA polymer membrane.

False color details of TEM images showing the external portion of (**A** and **B**) hybrid and (**C**) pristine polymersome membranes. No gaps or voids are detected between the gold (black) and PTA-stained polymer (orange to purple intensity gradient) phases. The images are represented using a *magma* colormap to enhance contrast.

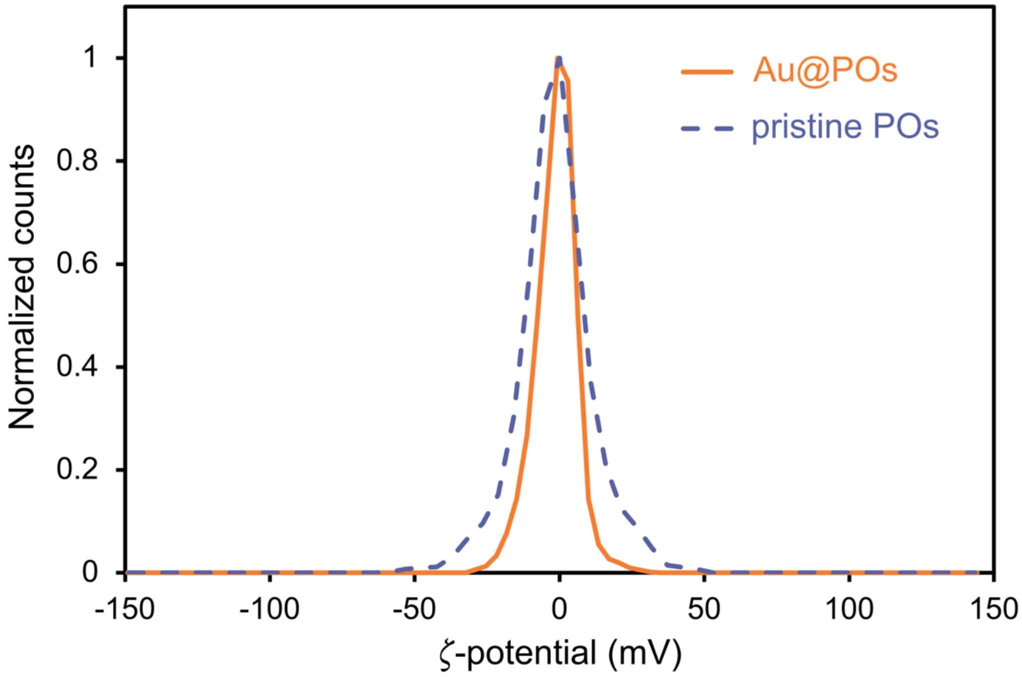

Figure S2. Surface potential characterization.

Normalized ζ-potential distributions of hybrid (Au@POs, orange solid line) and pristine polymersome dispersions (pristine POs, blue dashed line).

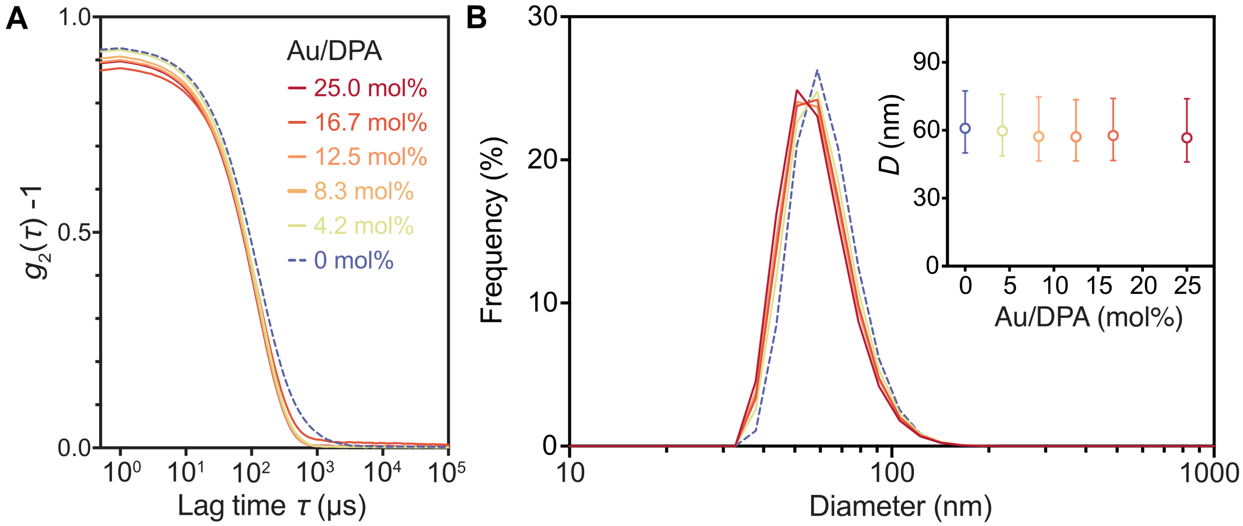

Figure S3. Size distributions of hybrid polymersome by dynamic light scattering (DLS).

(**A**) Representative DLS autocorrelation functions $g_2(\tau) - 1$ of hybrid polymersome dispersions synthesized at increasing Au/DPA molar percentages, compared to the pristine polymersomes (0 mol%). All the samples show a single exponential decay at comparable times. Each curve represents the averages of three measurements. (**B**) Number-averaged DLS size distributions, derived from the distribution analysis of the curves in (A), do not vary significantly after the *in situ* synthesis of gold nanoparticles up to 25 mol% Au/DPA. Shaded areas represent the standard deviation from triplicates. Each curve represents the average of three measurements. Inset: Averages (symbols) and spreads (error bars) of the diameters distributions as a function of the Au/DPA molar percentage.

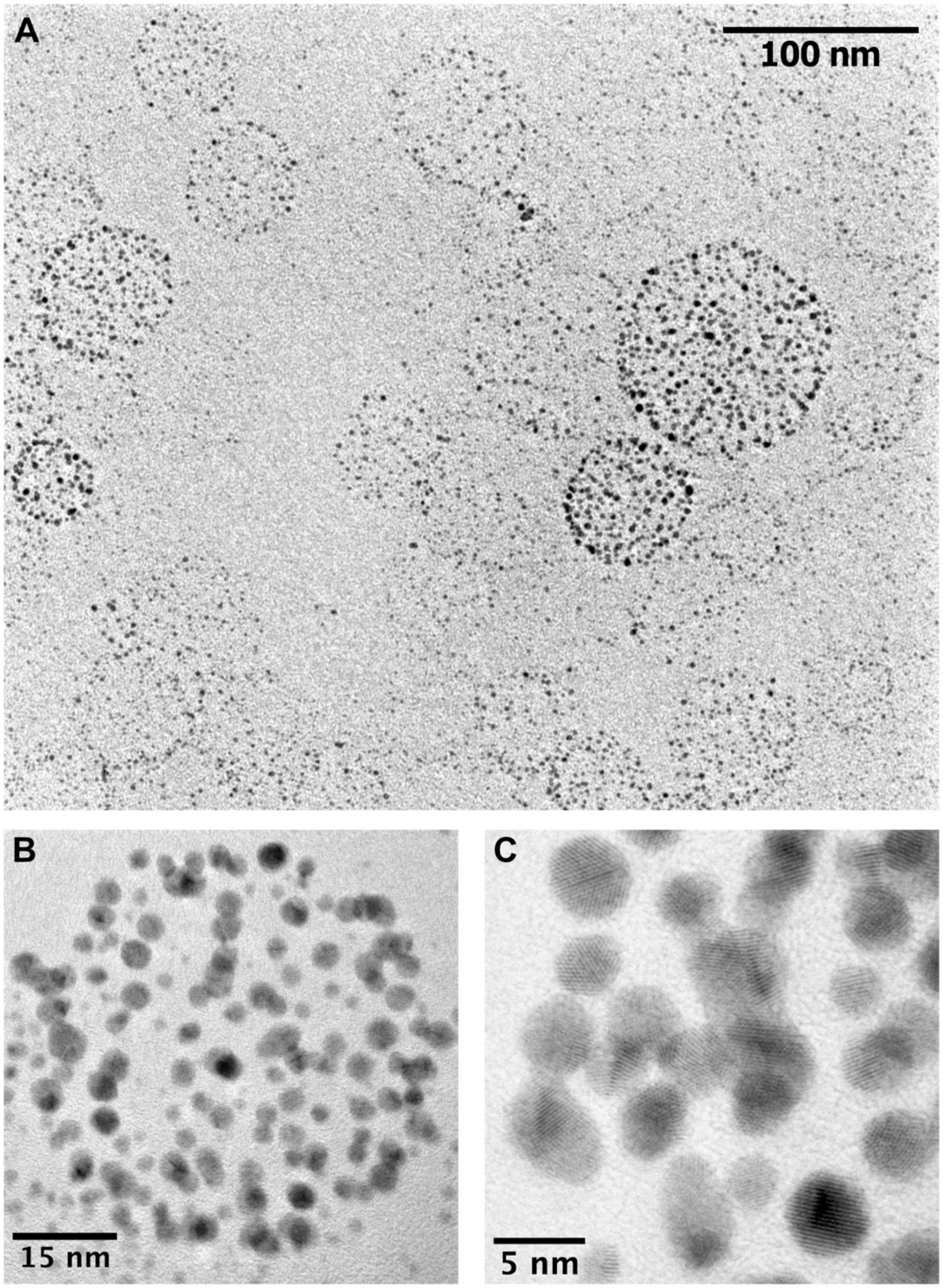

Figure S4. Unstained TEM images of hybrid polymersomes.

(**A**) Low-magnification image displaying gold nanoparticles arranged in random spherical arrays within hybrid polymersomes. (**B**) High-magnification image illustrating the distribution of gold nanoparticles within an individual polymersome. (**C**) High-magnification view of embedded gold nanoparticles, where the lattice planes are visible.

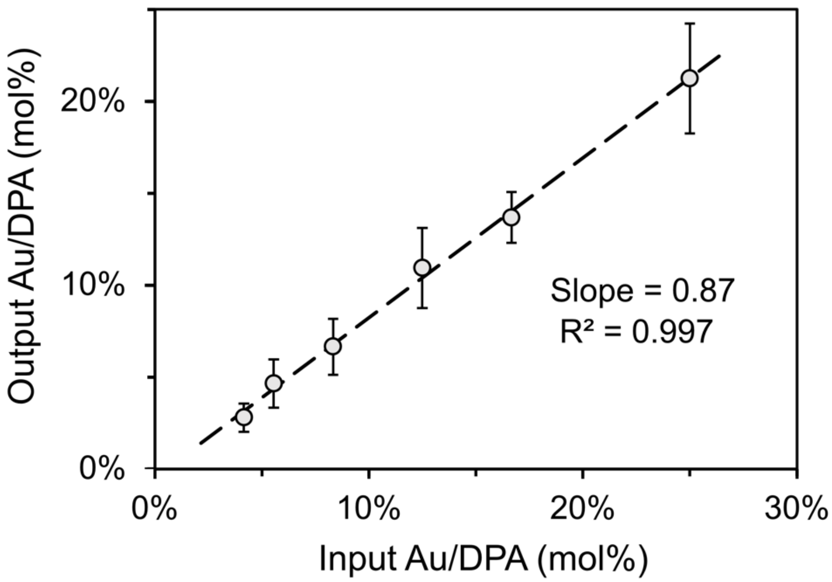

Figure S5. Evaluation of Au-loading yield.

Au/DPA molar percentage in 24 purified hybrid polymersome formulations (output) as a function of the Au/DPA molar percentages fed into the *in situ* reaction. Data points are obtained by averaging the concentration

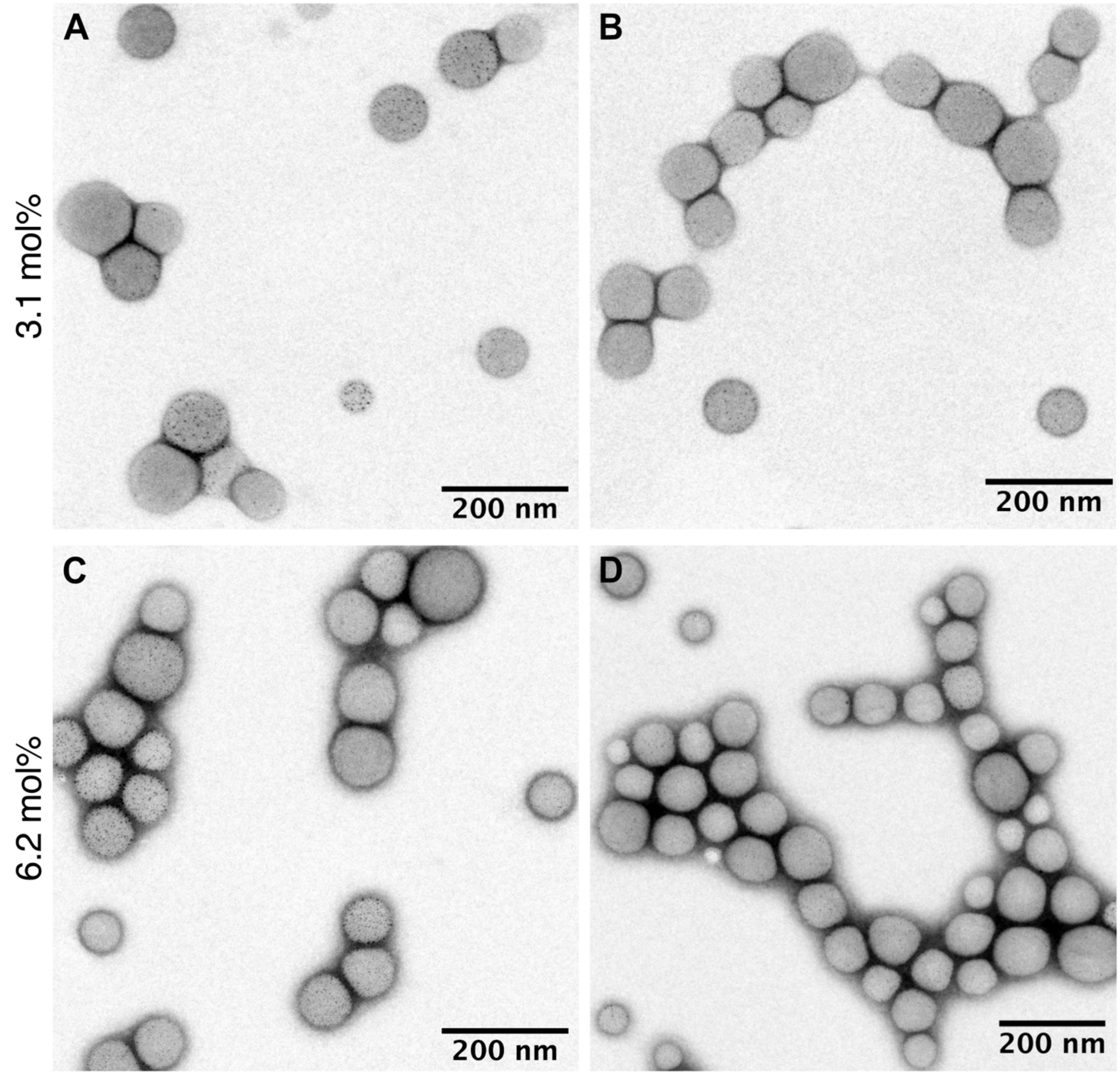

Figure S6. Representative TEM images of hybrid polymersome populations.

Low-magnification TEM images of hybrid polymersomes synthesized at Au/DPA ratios of (**A**, **B**) 3.1 mol%, and (**C**, **D**) 6.2 mol%.

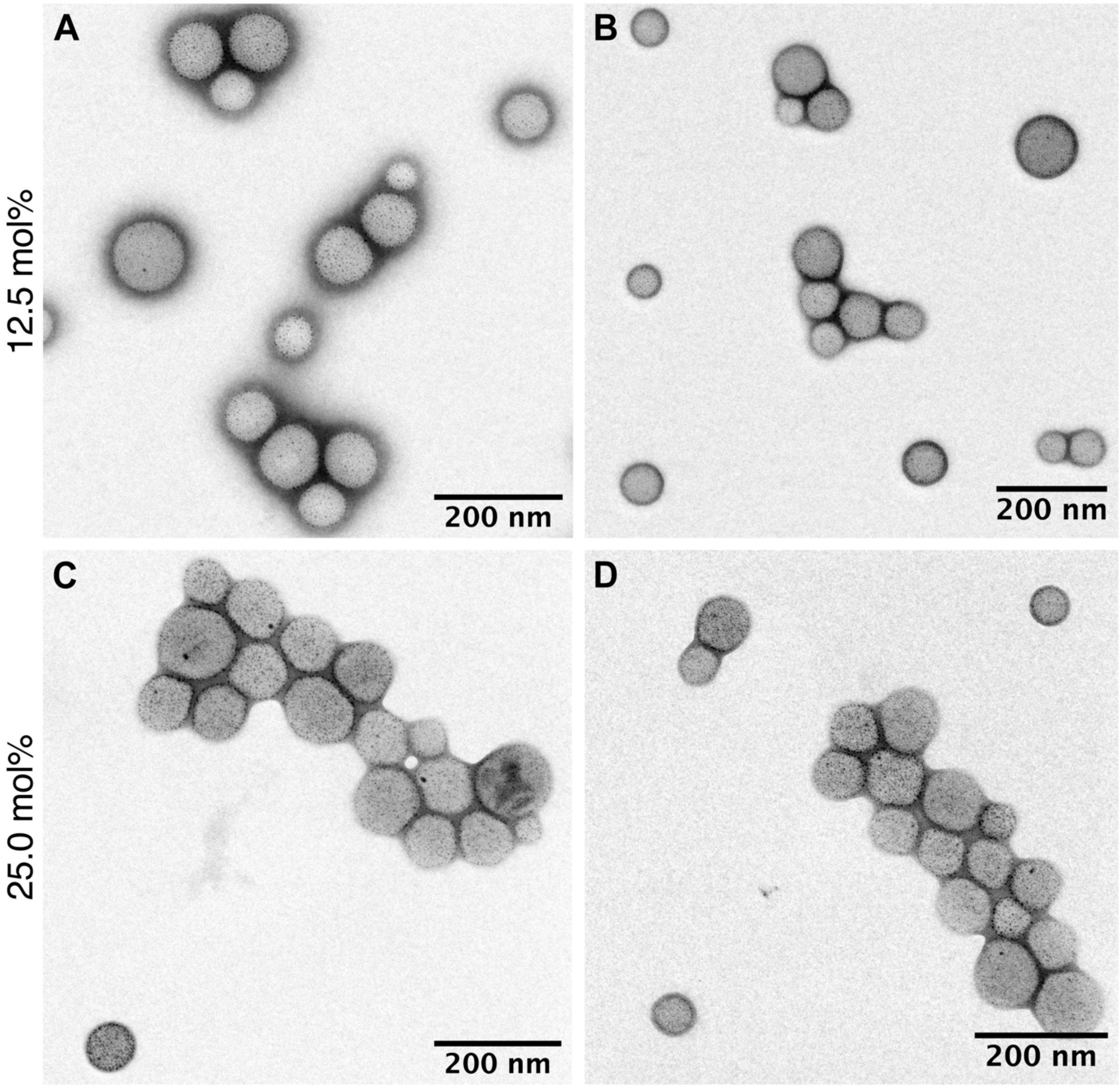

Figure S7. Representative TEM images of hybrid polymersome populations.

Low-magnification TEM images of hybrid polymersomes synthesized at Au/DPA ratios of (**A**, **B**) 12.5 mol%, and (**C**, **D**) 25 mol%.

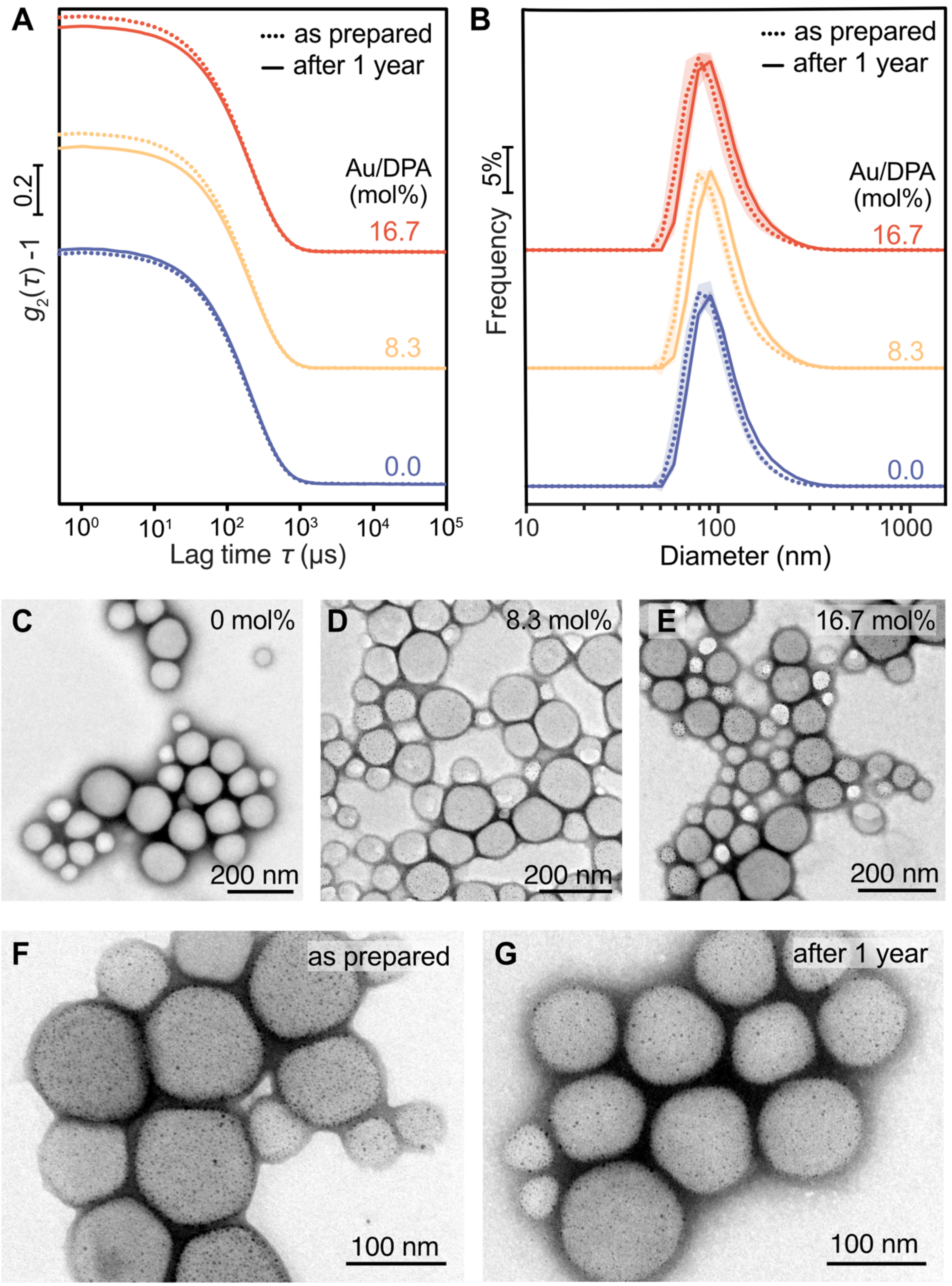
A
as prepared
after 1 year
Au/DPA (mol%)
16.7
8.3
0.0
0.2
$g_2(\tau)$ -1
10^0 10^1 10^2 10^3 10^4 10^5
Lag time τ (μs)
B
as prepared
after 1 year
Au/DPA (mol%)
16.7
8.3
0.0
5%
Frequency
10 100 1000
Diameter (nm)
C
0 mol%
200 nm
D
8.3 mol%
200 nm
E
16.7 mol%
200 nm
F
as prepared
100 nm
G
after 1 year
100 nm

Figure S8. Morphological characterization of the long-term stability of hybrid polymersomes.

(**A**) DLS autocorrelation functions and (**B**) number-averaged size distributions of pristine and hybrid polymersome dispersions measured after preparation (dotted lines) and after 1 year of storage at 4°C (solid lines). Shaded areas represent the standard deviation from triplicates. (**C** – **E**) Low-magnification TEM images displaying the populations of pristine (C) and hybrid polymersomes with 8.3 mol% (D) and 16.7 mol% (E) after one year at 4°C. (**F**, **G**) TEM images of 16.7 mol% hybrid polymersomes immediately after preparation (F) and after one year (G). The occasional bright patches result from electron beam damage during TEM and do not indicate sample degradation.

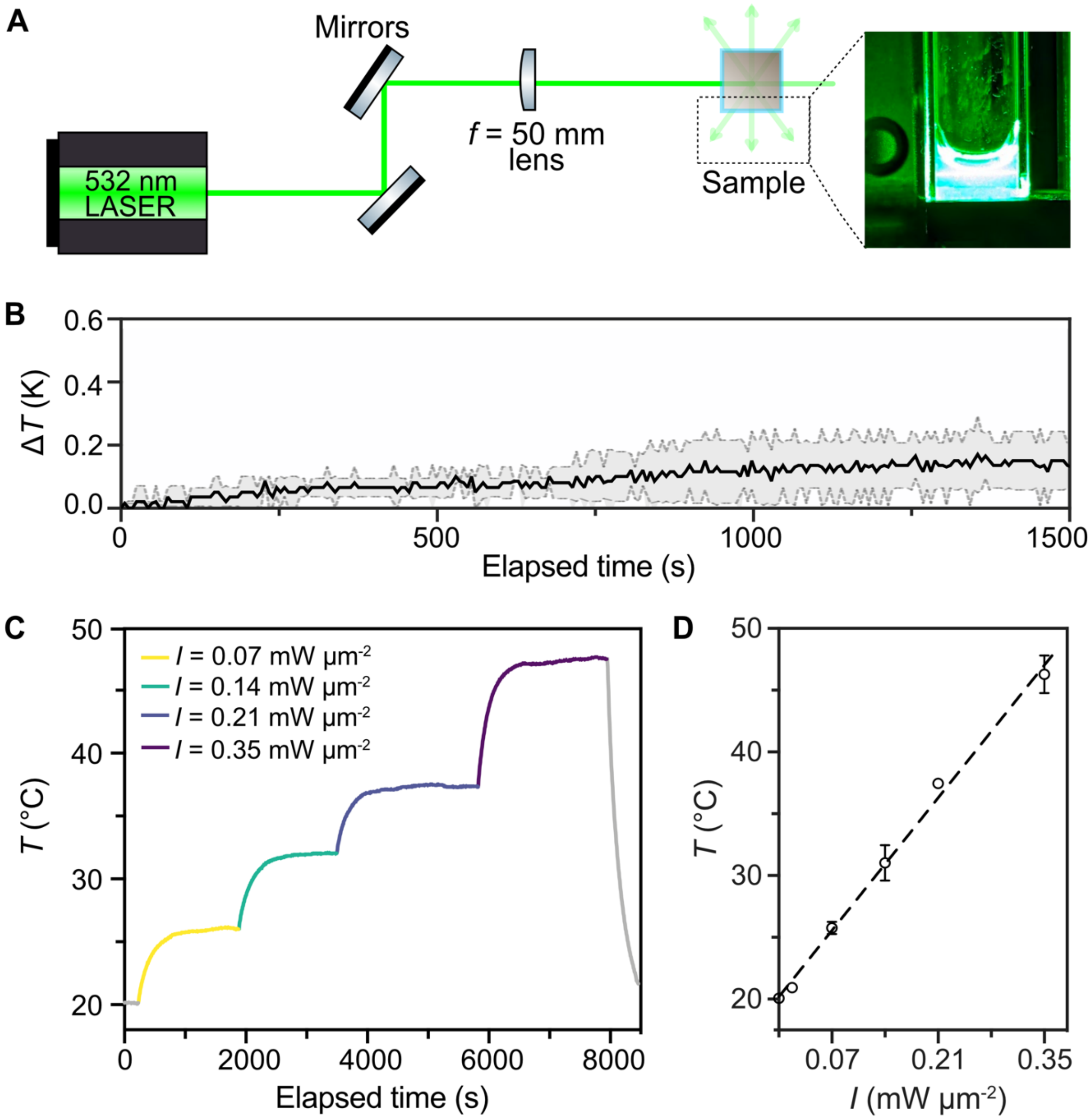

Figure S9. Characterization of hybrid polymersome thermoplasmonic properties.

(**A**) Scheme of the optical setup used for the thermoplasmonic characterization of hybrid polymersomes. Inset: photograph of a sample under laser irradiation. (**B**) Temperature increment measured in the optical setup shown in (A) when the cuvette is filled with PBS and irradiated with I = 0.128 mW µm^{-2}. (**C**) Thermal response of a ~ 60-nm hybrid polymersome dispersion (n = 9.6 Au@POs µm^{-3}, χ_{AuNP} = 109) irradiated at 532 nm with a stepwise increase of the incident laser intensity I. (**D**) The steady-state temperatures obtained from the previous plot depend linearly on the laser intensity.

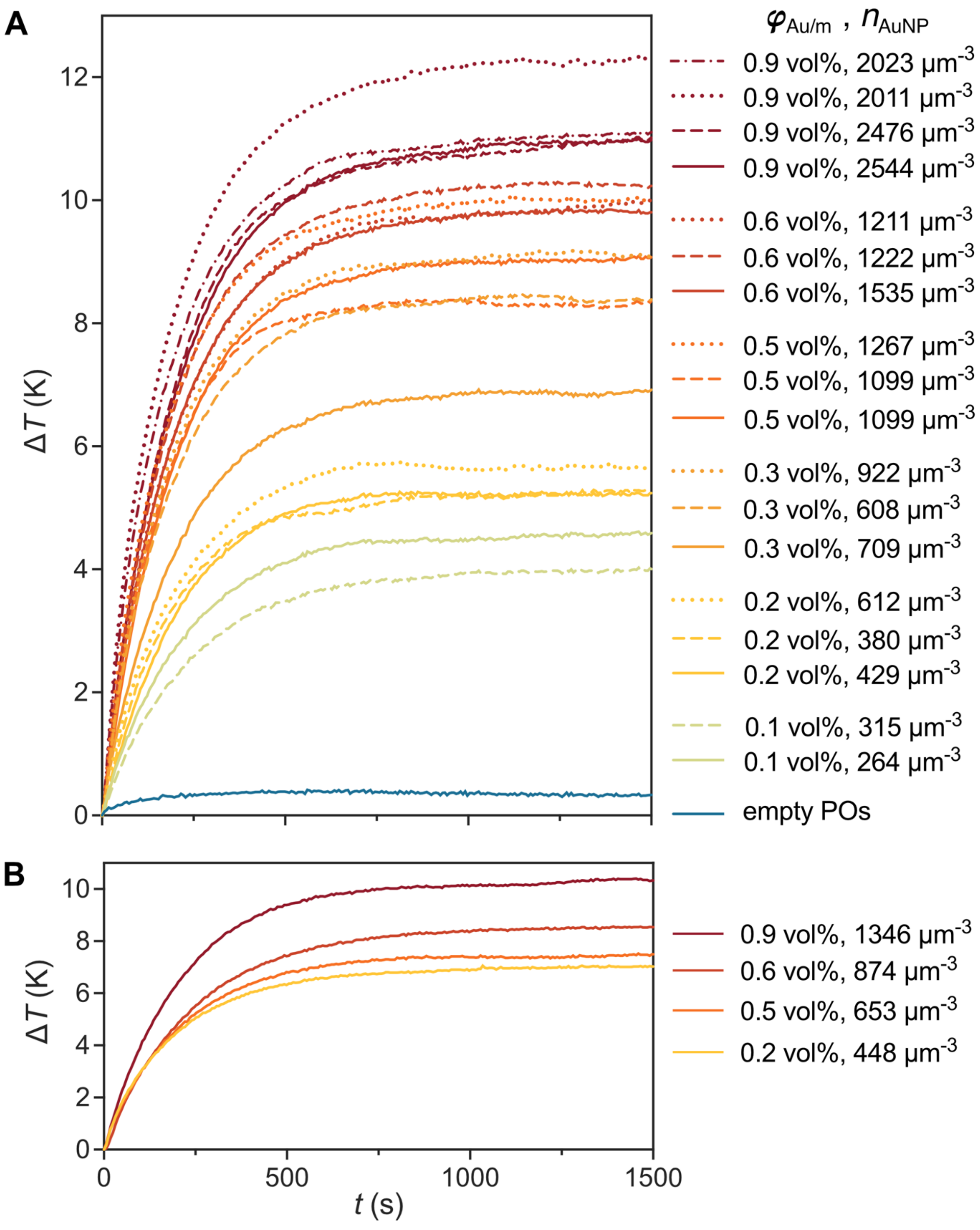

A
B
φAu/m , nAuNP
0.9 vol%, 2023 μm-3
0.9 vol%, 2011 μm-3
0.9 vol%, 2476 μm-3
0.9 vol%, 2544 μm-3
0.6 vol%, 1211 μm-3
0.6 vol%, 1222 μm-3
0.6 vol%, 1535 μm-3
0.5 vol%, 1267 μm-3
0.5 vol%, 1099 μm-3
0.5 vol%, 1099 μm-3
0.3 vol%, 922 μm-3
0.3 vol%, 608 μm-3
0.3 vol%, 709 μm-3
0.2 vol%, 612 μm-3
0.2 vol%, 380 μm-3
0.2 vol%, 429 μm-3
0.1 vol%, 315 μm-3
0.1 vol%, 264 μm-3
empty POs
ΔT (K)
0.9 vol%, 1346 μm-3
0.6 vol%, 874 μm-3
0.5 vol%, 653 μm-3
0.2 vol%, 448 μm-3
t (s)

Figure S10. ∆T-time traces for all the hybrid polymersome dispersion in the study.

Mean temperature-time evolution of each tested hybrid polymersome dispersion upon irradiation with a 532 nm laser of intensity of I = 0.128 mW μm^{-2}. (**A**) Formulations measured at a polymer concentration of 1 g/L. (**B**) Formulations measured at a polymer concentration of 0.67 g/L.

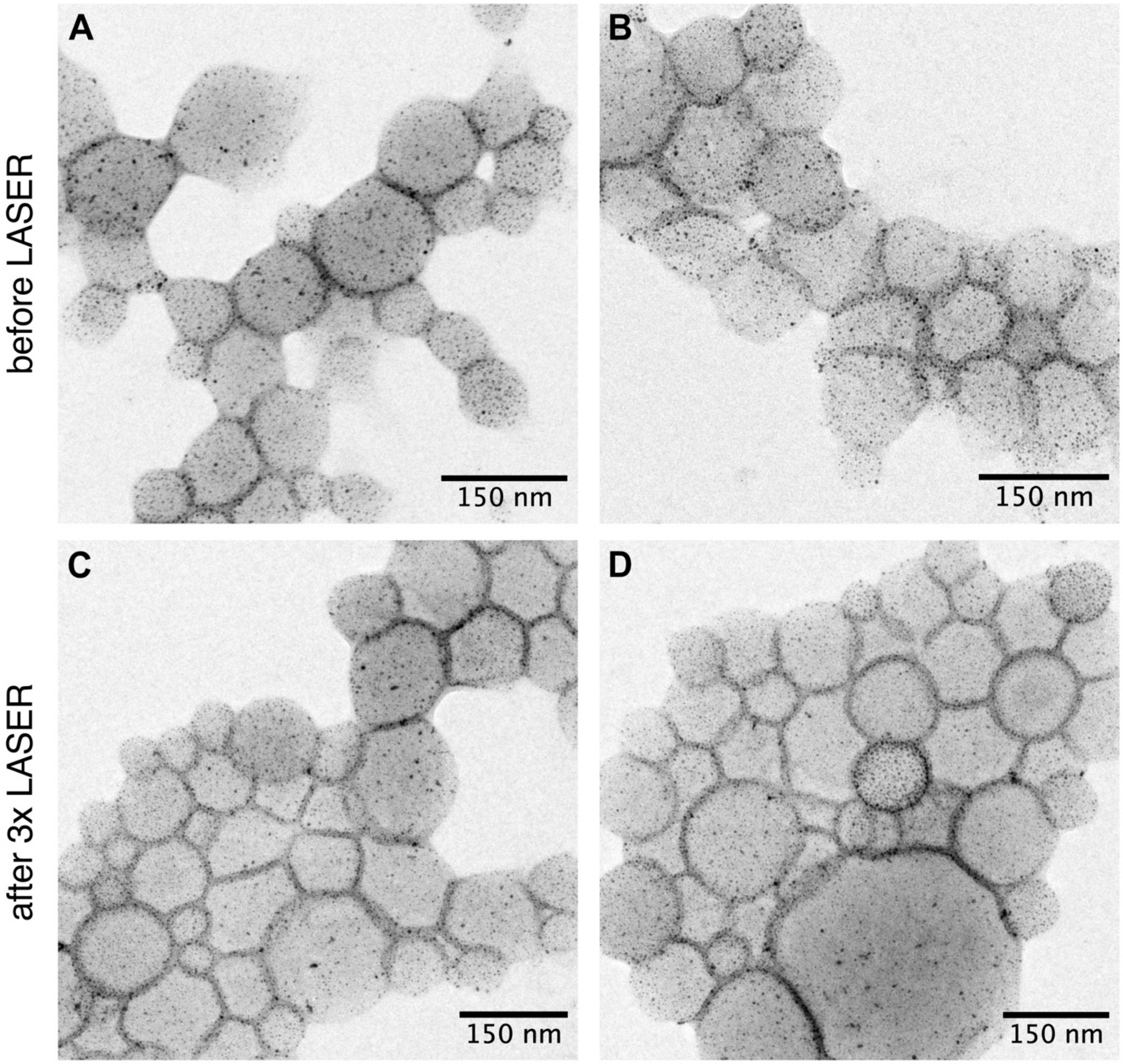

Figure S11. Thermoplasmonic heating does not affect polymersome integrity.

TEM images of 0.6 vol% hybrid polymersomes (**A**, **B**) before and (**C**, **D**) after three consecutive thermoplasmonic heating-cooling cycles, achieved by exposing the dispersion to a 532 nm laser with an intensity of $I = 0.128$ mW µm^{-2} for 1200 s (peak $\Delta T = 12$ K), and subsequently removing the laser stimulus until the temperature dropped to room temperature.

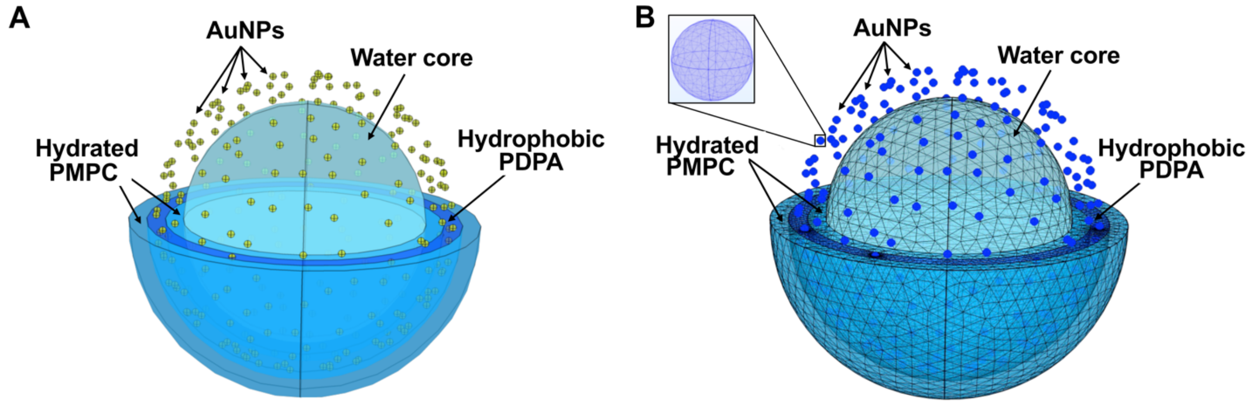

Figure S12. Computational model of the hybrid polymersome.

(**A**) Model of the hybrid polymersome used for the calculation of the optical properties by Mie theory. (**B**) The meshing applied to the hybrid polymersome model represented in (A) for the finite element simulation of the thermoplasmonic response in Comsol Multiphysics.

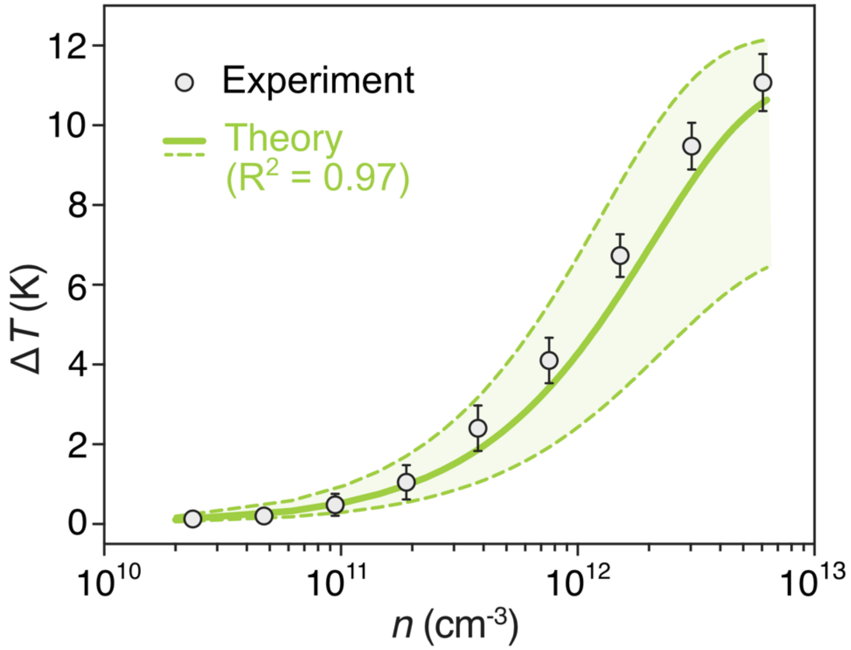

Figure S13. Representation of Figure 3D as a function of *n*.

Experimental steady-state temperature increments ΔT (circles) in a progressively diluted dispersion of ~100-nm hybrid polymersomes ($\varphi_{\mathrm{Au/m}} = 0.9$ vol%, $\chi_{\mathrm{AuNP}} = 262$) exposed to a 532 nm laser with intensity $I = 0.128$ mW μm^{-2} as a function of the polymersome number density n. Data points are the mean of three measurements, and error bars represent the standard deviation. Theoretical predictions are represented as solid green lines. The shaded area delimited by the dashed green lines is the confidence interval for the prediction calculated over the experimental size distributions of gold nanoparticles and polymersomes.

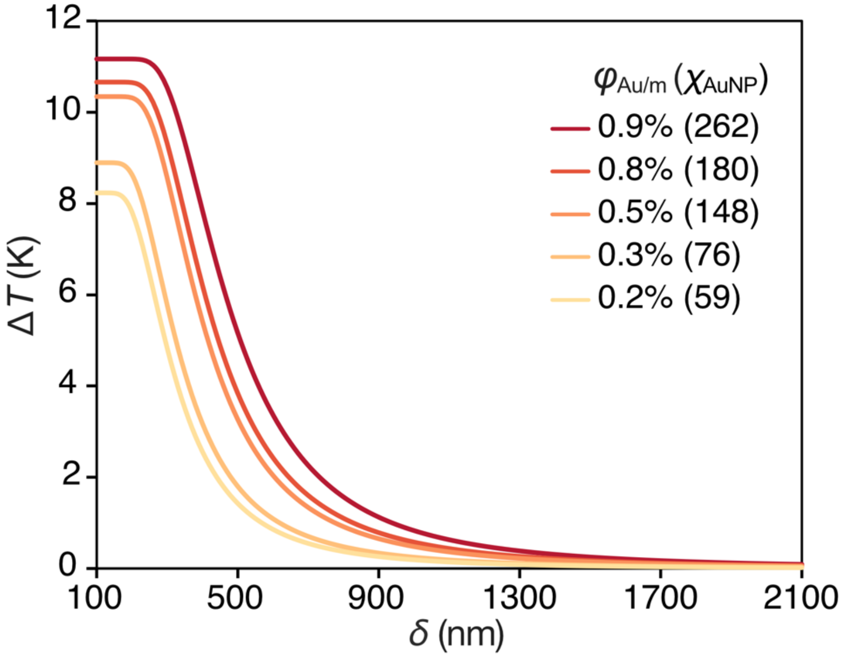

Figure S14. Dependency of thermoplasmonic heating on spacing and Au-loading.

Theoretical temperature increments ΔT of $\sim$100-nm hybrid polymersomes at different Au-loadings as a function of the mean inter-polymersome spacing δ upon exposure to a 532 nm laser with intensity $I = 0.128$ mW μm^{-2}.

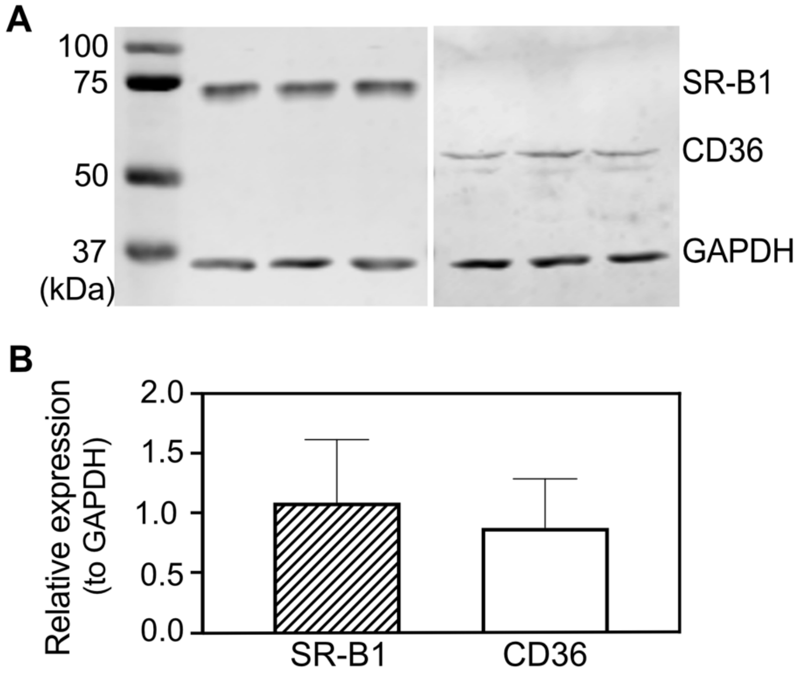

Figure S15. Evaluation of receptor expression in T98G cells.

(**A**) Representative images of Western Blot membranes obtained from T98G cell lysates revealing the expression of SR-B1 (61-82 kDa, left) and CD36 (53 kDa, right) receptors, compared to the GAPDH (37 kDa) loading control. (**B**) Quantification of the relative expression of the two receptors compared to GAPDH.

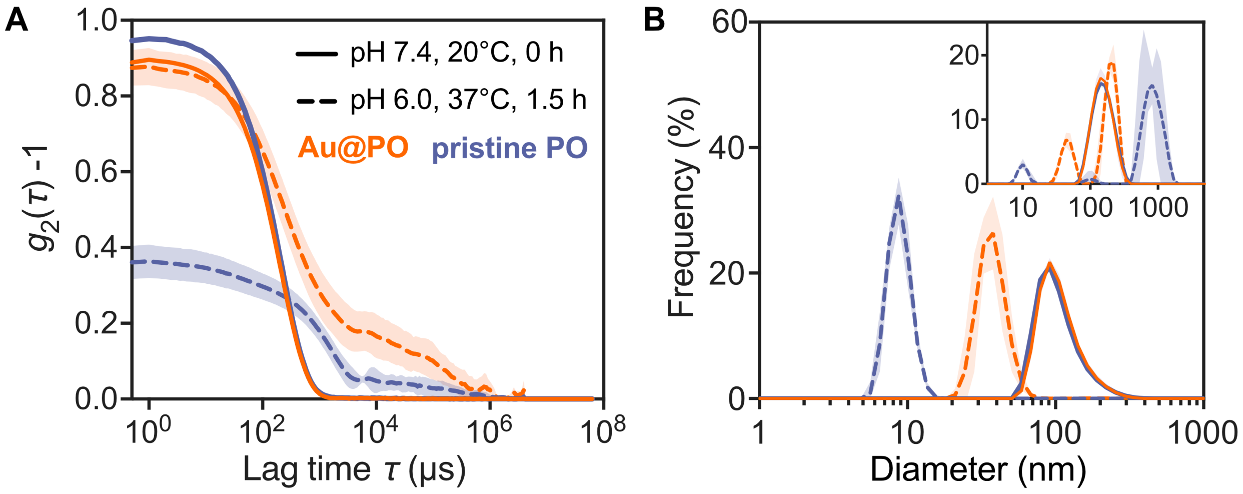

Figure S16. Degradation study of hybrid and pristine polymersomes at the endosomal pH and temperature.

(**A**) DLS autocorrelation functions $g_2(\tau) - 1$ of pristine (blue) and hybrid polymersome (orange) dispersions at pH 7.4 and 20 °C (solid line) and after 1.5 h at pH 6.0 and 37 °C (dashed lines). (**B**) DLS number-averaged distribution calculated by distribution analysis of the correlation functions in (A). The intensity-averaged distribution, revealing the formation of a few large aggregates in the degraded polymersome dispersions, is shown in the inset. The shaded areas in both (A) and (B) correspond to the standard deviation of three repeated measures.

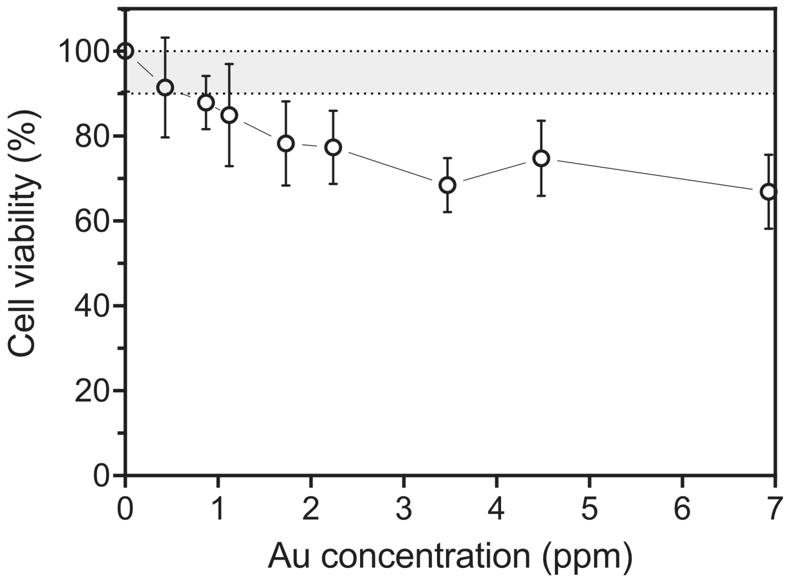

Figure S17. Intrinsic cytotoxicity of hybrid polymersomes and assessment of a safe dose.

T98G cells' viability, as evaluated by colorimetric MTT metabolic assay, after 1.5 h of incubation with hybrid polymersomes. The shaded area represents the non-toxic region with > 90% viability.

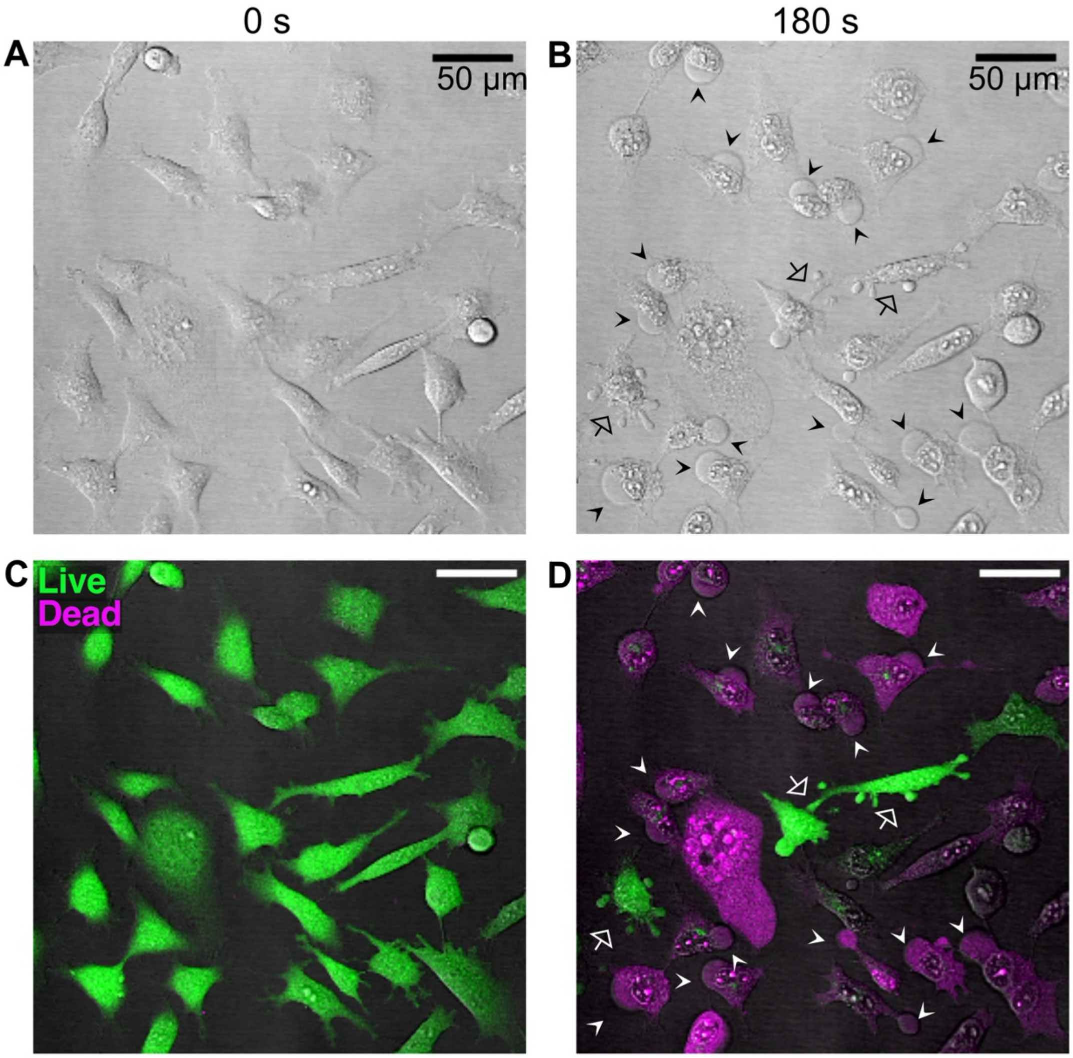

Figure S18. Identification of morphological markers for cell death mechanism assessment.

(**A**, **B**) Bright-field and (**C**, **D**) live/dead stained fluorescence microscopy composite images of T98G cells treated with hybrid polymersomes (**A**, **C**) before and (**B**, **D**) after 180 s of cumulative exposure to the 514 nm laser at intensity $I = 0.8$ mW μm^{-2}. Empty triangular arrowheads indicate episodes of blebbing and formation of apoptotic bodies, while filled barbed arrowheads indicate leakage of cytosolic genetic material.

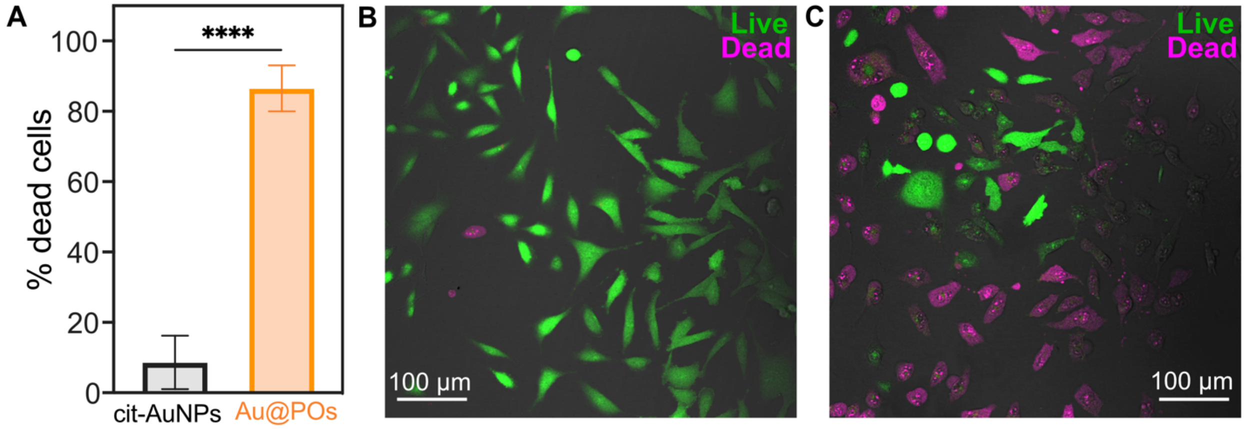

Figure S19. Performance of hybrid polymersomes vs non-targeting gold nanoparticles in plasmonic hyperthermia.

(**A**) The plasmonic-induced mortality of T98G cells treated with hybrid polymersomes (Au@POs, orange) is significantly higher than in cells treated with a 2-fold higher dose of citrate-capped gold nanoparticles (cit-AuNPs, grey). Error bars are the standard deviations of three (cit-AuNPs) and seven (Au@POs) independent experiments. Statistical significance was evaluated by Student's t-test (**** $p \leq 0.0001$) (**B**-**C**) Representative composite confocal micrographs recorded after 3 minutes of cumulative 514nm-laser irradiation of live/dead stained T98G cells treated with (B) citrate-capped AuNPs, [Au] = 0.8 ppm, and (C) hybrid polymersomes, [Au] = 0.4 ppm.

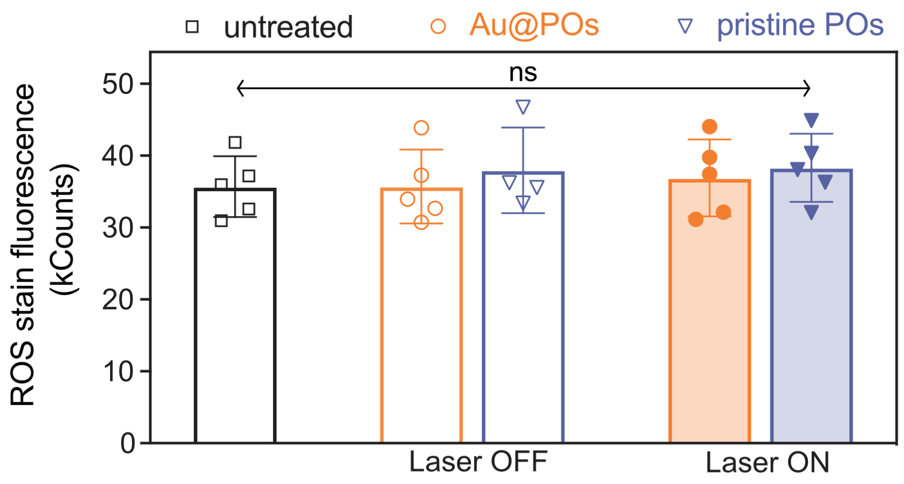

Figure S20. Plasmonic hyperthermia with hybrid polymersomes does not induce significant reactive oxygen species (ROS) evolution.

Reactive oxygen species (ROS) evolution measured by fluorescent assay on T98G cells treated with both hybrid polymersomes (Au@POs, orange circles) and pristine POs (blue inverted triangles) showed no significant difference compared to untreated cells (black squares), whether the laser was ON or OFF. Data points represent independent experiments, columns represent means, and error bars are standard deviations. Statistical significance was evaluated by two-way ANOVA (ns: $p > 0.05$).

SUPPORTING VIDEOS

Movie S1.

Visual demonstration by infrared imaging of the thermoplasmonic response of a hybrid polymersome (Au@PO) dispersion to 532 nm laser exposure.

Movie S2.

Live/dead confocal microscopy imaging of T98G cells treated with hybrid polymersomes and exposed to LSPR laser excitation (514 nm, I = 0.8 mW µm-2). The timestamp shows the exposure time to the LSPR excitation laser. Live and dead cells are labeled in green and magenta, respectively.

Movie S3.

Live/dead confocal microscopy imaging of T98G cells treated with hybrid polymersomes without LSPR laser excitation (laser OFF condition). The timestamp shows, for comparison, the equivalent exposure time to the LSPR excitation laser (514 nm) experienced by cells treated in the laser ON condition. Live and dead cells are labeled in green and magenta, respectively.

Movie S4.

Live/dead confocal microscopy imaging of T98G cells treated with pristine polymersomes and exposed to laser excitation (514 nm, I = 0.8 mW µm-2). The timestamp shows the exposure time to the LSPR excitation laser (514 nm). Live and dead cells are labeled in green and magenta, respectively.

REFERENCES

(1) Baffou, G.; Quidant, R.; Abajo, F. J. G. D. Nanoscale Control of Optical Heating in Complex Plasmonic Systems. *ACS Nano* **2010**, *4*, 709–716. https://doi.org/10.1021/nn901144d.

(2) Nield, D. A.; Bejan, A. *Convection in Porous Media*; Springer New York, 2012.

(3) Han, J. C. *Analytical Heat Transfer*; CRC Press, 2016.

(4) Tournus, F. Random Nanoparticle Deposition: Inter-Particle Distances in 2D, 3D, and Multilayer Samples. *Journal of Nanoparticle Research* **2011**, *13*, 5211–5223. https://doi.org/10.1007/s11051-011-0506-9.
